# Bio‐Plausible Multimodal Learning with Emerging Neuromorphic Devices

**DOI:** 10.1002/advs.202406242

**Published:** 2024-09-11

**Authors:** Haonan Sun, Haoxiang Tian, Yihao Hu, Yi Cui, Xinrui Chen, Minyi Xu, Xianfu Wang, Tao Zhou

**Affiliations:** ^1^ School of Automation Engineering University of Electronic Science and Technology of China Chengdu 611731 China; ^2^ State Key Laboratory of Electronic Thin Film and Integrated Devices University of Electronic Science and Technology of China Chengdu 611731 China

**Keywords:** multifunctional integration, multimodal learning, multiterminal device, neuromorphic computing

## Abstract

Multimodal machine learning, as a prospective advancement in artificial intelligence, endeavors to emulate the brain's multimodal learning abilities with the objective to enhance interactions with humans. However, this approach requires simultaneous processing of diverse types of data, leading to increased model complexity, longer training times, and higher energy consumption. Multimodal neuromorphic devices have the capability to preprocess spatio‐temporal information from various physical signals into unified electrical signals with high information density, thereby enabling more biologically plausible multimodal learning with low complexity and high energy‐efficiency. Here, this work conducts a comparison between the expression of multimodal machine learning and multimodal neuromorphic computing, followed by an overview of the key characteristics associated with multimodal neuromorphic devices. The bio‐plausible operational principles and the multimodal learning abilities of emerging devices are examined, which are classified into heterogeneous and homogeneous multimodal neuromorphic devices. Subsequently, this work provides a detailed description of the multimodal learning capabilities demonstrated by neuromorphic circuits and their respective applications. Finally, this work highlights the limitations and challenges of multimodal neuromorphic computing in order to hopefully provide insight into potential future research directions.

## Graphical abstract

Multimodal neuromorphic computing strives to achieve enhanced biologically plausible multimodal learning with low complexity and high energy‐efficiency. This review provides a comprehensive overview of bio‐plausible multimodal learning with emerging neuromorphic devices, specifically focusing on multimodal neuromorphic computing and their capabilities in processing multiple physical signals, coupled with a forward‐looking perspective of multimodal neuromorphic computing.

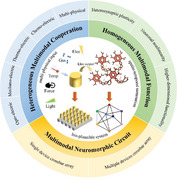

## 1 Introduction

The implementation of artificial intelligence using Von Neumann machines has brought convenience to human beings,^[^ ^1^, ^2^, ^3^, ^4^, ^5^ ^]^ but it also poses limitations on wider application due to the huge energy consumption resulting from the separation of memory and data processing units, commonly referred to as the memory wall.^[^ ^6^ ^]^ Inspired by the powerful functionalities and high energy‐efficiency of human brain,^[^ ^7^, ^8^, ^9^, ^10^, ^11^, ^12^ ^]^ neuromorphic computing has been proposed to simulate synapses, neurons, as well as the distributed architecture of the brain,^[^ ^9^, ^10^ ^]^ in order to develop low‐power parallel processing systems for artificial intelligence.^[^ ^11^, ^12^, ^13^, ^14^ ^]^ Since Carver Mead^[^ ^15^ ^]^ first proposed the utilization of device physical properties for the implementation of neuromorphic systems in 1900, complementary metal oxide semiconductor transistors (CMOS)^[^ ^16^ ^]^ and a range of devices with memristive properties, including phase change memory (PCM),^[^ ^17^ ^]^ resistive random access memory (RRAM),^[^ ^18^ ^]^ electrochemical random access memory (ECRAM),^[^ ^19^ ^]^ magnetic random access memory (MRAM),^[^ ^20^ ^]^ ferroelectric random access memory (FeRAM),^[^ ^21^ ^]^ etc., have been employed to replicate synaptic^[^ ^22^, ^23^, ^24^ ^]^ and neuronal^[^ ^25^, ^26^, ^27^ ^]^ functions. However, the majority of devices oversimplify synaptic and neuronal functions to merely storing weights and integrate‐and‐fire function,^[^ ^28^, ^29^, ^30^, ^31^, ^32^, ^33^ ^]^ disregarding the brain's perceptual capabilities and microscopic neural processes, thereby constraining further development of neuromorphic computing.

The brain is capable of developing a comprehensive understanding of the real world and acquiring intricate rules through various specific channels of sensory input, known as multiple modalities.^[^ ^34^, ^35^, ^36^, ^37^, ^38^ ^]^ Specifically, the integration of multimodal information from the similar or related sources is utilized to enhance comprehension (modal integration) and learn different expressions of the same concept (modal transfer). In this process, sensory neurons integrate different modalities of information into unified electrical pulses those are transmitted the brain. Subsequently, interconnected neurons in various regions of the brain are activated sequentially to extract the correlated information and achieve a comprehensive understanding of stimuli.^[^ ^39^, ^40^, ^41^ ^]^ Motivated by the multimodal learning capabilities of the brain, a framework for multimodal machine learning has been proposed to implement multimodal information processing similar to the brain,^[^ ^42^, ^43^ ^]^ which enhances human‐machine interaction in areas such as information retrieval,^[^ ^44^ ^]^ machine translation^[^ ^45^ ^]^ and mobile identity authentication.^[^ ^46^ ^]^ However, this framework requires various sensor arrays for perception in order to convert a wide range of physical signals into digital signals with distinctive data types. Additionally, it fully leverages software to simulate multi‐connected neuronal networks for analysis, thereby leading to an increase in hardware overhead, software complexity, and power consumption. To address this problem, certain emerging neuromorphic devices are being utilized to further simulate the mechanism and function of organisms, with the aim of achieving low‐complexity and energy‐efficient bio‐plausible multimodal learning.

**Figure 1:**
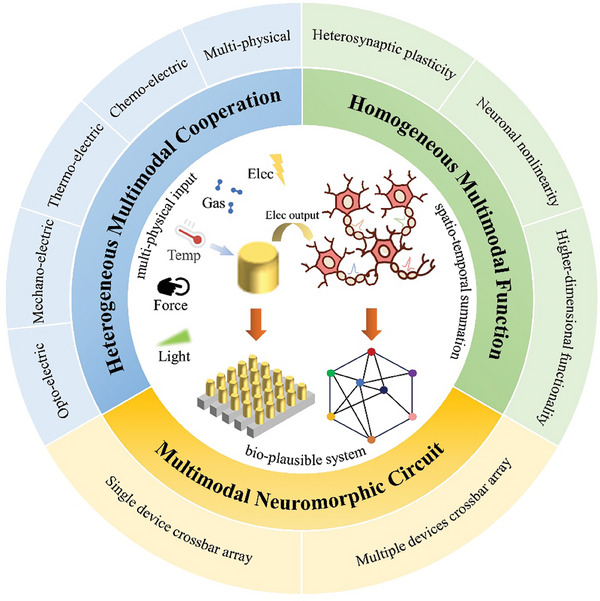
The summarization of multimodal neuromorphic computing. Heterogeneous multimodal neuromorphic devices are modulated by different physical signals, which can be divided into opto‐electric, mechano‐electric, thermo‐electric, and chemo‐electric cooperation according to the signal type. Homogeneous multimodal neuromorphic devices are modulated by multiple same‐physical signals, which are divided into electrical and optical sections, emphasizing the use of a single device to implement complex bioinspired computations. The multimodal neuromorphic circuit is the integration of individual devices to achieve fine‐grained sensing and higher dimensional computations, including image recognition, inference, etc.

One aspect of these neuromorphic devices is dedicated to simulating various sensory modalities found in organisms, such as vision,^[^ ^47^, ^48^, ^49^ ^]^ touch,^[^ ^50^ ^]^ olfaction,^[^ ^51^ ^]^ thermoreception,^[^ ^52^ ^]^ and humidity sensing.^[^ ^53^ ^]^ The goal is to detect diverse physical signals and concurrently preprocess the spatio‐temporal information into a unified electrical signal, with the aim of enabling multi‐modal perception on a single device^[^ ^54^, ^55^ ^]^ (e.g., simulating skin for sensing both temperature and pressure^[^ ^56^ ^]^). The other aspect of these neuromorphic devices emphasizes mimicking the architecture and functionality of multi‐connection neurons,^[^ ^57^ ^]^ to simultaneously process multiple signals carrying distinct information. Each of these devices has the capability to process multiple physical signals and integrate spatio‐temporal information into a unified signal, thus holding the potential to facilitate bio‐plausible multimodal learning, which is referred to as multimodal neuromorphic devices in this review. **Figure** **1** provides an overview of the research areas of interest in multimodal neuromorphic computing, including heterogeneous and homogeneous multimodal devices, as well as multimodal neuromorphic circuits. We aim to present a comprehensive review on the achievement of bio‐plausible multimodal learning on neuromorphic hardware platforms, a topic not previously covered in existing review articles, and hopefully promote further development in this area in order to realize the widespread application of multimodal learning systems, particularly within the context of multisensory human‐machine interaction platform.^[^ ^58^ ^]^

In this review, we initially compare the expressions of multimodal machine learning and multimodal neuromorphic computing. Subsequently, we categorize multimodal neuromorphic devices into heterogeneous and homogeneous types based on the physical signals and provide detailed descriptions for each. In the section on heterogeneous multimodal neuromorphic devices, our focus lies in achieving the perception and processing of multiple physical signals based on their working principles. The section on homogeneous multimodal neuromorphic devices examines the role of these devices in simulating multi‐connected neurons through modulation by multiple identical physical signals. Afterward, we will delve into the functionalities and applications of multimodal neuromorphic circuits. Finally, we discuss the prospects for the limitations, challenges, and advancements in multimodal neuromorphic computing.

## 2 Expression of Multimodal Learning

**Figure 2:**
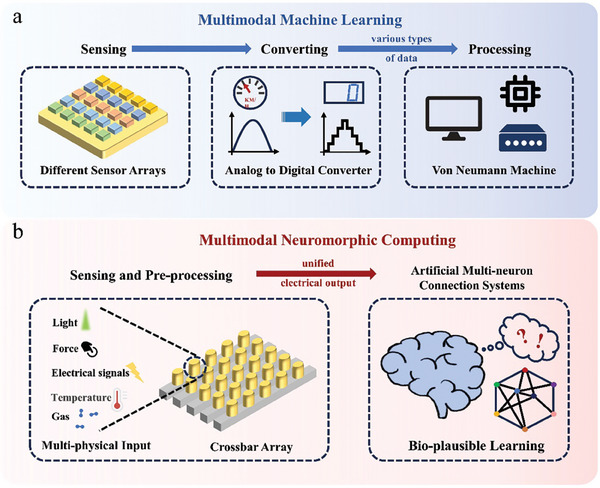
Expressions of Multimodal Learning. a) Training of a multimodal machine learning model using a von Neumann machine. Different sensors in the sensor array sense diverse physical signals, which are then converted by analog‐to‐digital converters into data with different types for input into the von Neumann machine to train the neural network model. b) Simple schematic of the multimodal neuromorphic system. Heterogeneous multimodal neuromorphic devices with multi‐physics sensing ability are connected into crossbar arrays to sense different physical signals and pre‐process the spatio‐temporal information. Then the processed information is fed into the system formed by the connection of homogeneous multimodal neuromorphic devices to realize bio‐plausible learning.

Multimodal learning tasks can be categorized into heterogeneous and homogeneous types based on variations in the perceived physical signals or data types. For instance, the utilization of video and audio signals for speech recognition is referred to as heterogeneous multimodal learning,^[^ ^59^ ^]^ while recognition of objects using different features within the same image is classified as homogeneous multimodal learning.^[^ ^60^ ^]^ In multimodal machine learning, multiple sensors are utilized to acquire external physical signals. These signals are then transformed into information with diverse data structures, and then processed using a von Neumann machine to train a deep learning model (**Figure** **2**a).^[^ ^61^ ^]^ In accordance with the brain's capacity for multimodal learning, encompassing modal integration and transfer, multimodal machine learning can be further delineated into the following five components: representation, alignment, fusion, translation, and co‐learning.^[^ ^42^ ^]^ Representation refers to the enhancement of feature expressions through the utilization of synergies across multiple modalities and reduction of redundancies within modalities, including joint representations^[^ ^44^ ^]^ and coordinated representations.^[^ ^62^ ^]^ Alignment means the process of identifying the correspondence between sub‐branches or elements of different modal information within a single instance, encompassing both temporal sequence alignment^[^ ^63^ ^]^ and spatial alignment.^[^ ^64^ ^]^ Fusion involves integrating data from various sources to enhance target prediction accuracy, such as video^[^ ^65^ ^]^ and audio^[^ ^66^ ^]^ recognition, multimodal sentiment analysis,^[^ ^67^ ^]^ and mobile identity authentication.^[^ ^46^ ^]^ Translation, also known as mapping, is the transformation of information from one modality to another, and is used for applications including machine translation,^[^ ^45^, ^68^, ^69^ ^]^ picture description,^[^ ^70^ ^]^ video description,^[^ ^71^ ^]^ and speech synthesis.^[^ ^72^ ^]^ Co‐learning refers leveraging information from a resource‐rich modality to support the learning of a relatively resource‐poor modality, and can be categorized into the areas of transfer learning,^[^ ^73^ ^]^ zero‐sample learning,^[^ ^74^ ^]^ single‐sample learning,^[^ ^75^ ^]^ co‐training,^[^ ^76^ ^]^ and so on. Among these aspects, the most crucial is the characterization and alignment of different modal information with uniform rules. Only after this can the fusion of modal integration occurs, as well as the translation and co‐learning associated with modal transfer.

In recent years, researchers have increasingly utilized neuromorphic systems instead of von Neumann machines for the training of deep learning models,^[^ ^77^, ^78^ ^]^ such as leveraging matrix‐vector multiplication with memristive crossbar arrays to accelerate computation,^[^ ^79^ ^]^ or employing interconnected neuron networks and spike learning rules to simulate biological learning processes.^[^ ^80^ ^]^ This approach has the potential to enhance computing speed and recognition accuracy, while also reducing the software overhead.^[^ ^81^ ^]^ However, it still relies on discrete sensor arrays for information sensing, necessitating additional design of sensor connections to process the spatio‐temporal information. Furthermore, it does not fundamentally address the fundamental challenge of multimodal learning.

To solve these problems, the researchers are endeavoring to utilize the physical properties of the device for analog computation of multiple physical signals, so as to achieve a unified representation with multiple modalities, spatio‐temporal data pre‐processing, as well as powerful regulation and rich functionality.^[^ ^51^, ^82^, ^83^, ^84^, ^85^, ^86^ ^]^ For example, Zhu et al.^[^ ^56^ ^]^ utilize artificial spiking neurons to sense pressure and temperature simultaneously, and then integrate them into electrical spiking signals, simplifying the subsequent information extraction and processing process, improving the accuracy of image recognition. In this review, the devices with such physical properties are referred as multimodal neuromorphic devices, which can be modulated by multiple physical signals simultaneously, store these physical signals in internal state variables, and utilize their correlations to achieve bio‐plausible brain‐like multimodal learning (Figure 2b). Multimodal neuromorphic devices can be further categorized into heterogeneous^[^ ^51^, ^82^, ^83^, ^84^, ^85^, ^86^ ^]^ and homogeneous multimodal neuromorphic devices^[^ ^87^, ^88^, ^89^ ^]^ according to the physical characteristics of the signals. Generally, the former refers to the devices those receive diver types of physical signals, while the latter are associated with those receiving homogeneous types of physical signals. Both types of devices feature multiple input terminals, including actual terminals consisting of metal electrodes and virtual terminals encompassing the modulation of materials by physical signals. Each of the terminals can modulate the internal state variables of devices, resulting the differences in output properties. Here we pick some representative works as listed in **Table** **1**. Some examples of multimodal neuromorphic devices are given below, to distinguish them from conventional multiterminal devices.

**Table 1:** Summary of bio‐plausible multimodal learning using emerging devices.

Type of Device	Type of Input Signals	Materials	Function	Power Consumption	Response time	Type of task	Accuracy	Refs.
Heterogeneous Multimodal Neuromorphic Device	Optical and electrical	MoS_2_	Metaplasticity	4.8*pJ*	*≈ms*	Classical conditioning	*N/A*	[90]
		MoS_2_	Scotopic and photopic adaptation	≈*µJ*	*≈s*	Accurate perception	97%	[91]
		MoO_x_	Light‐tunable plasticity and pre‐processing	*N/A*	*≈ms*	Inage recognition	99%	[92]
		α‐In_2_Se_3_	Adjustable nonlinear transformation and multisensory fusion	≈*nJ*	*≈ms*	Handwritten patterns recognition (MNIST)	85%	[93]
		VO_2_	Sensing, memory and pre‐process	≈*nJ*	*≈s*	Handwritten patterns recognition (MNIST)	93%	[94]
	Mechanical and electrical	AgNWs/PDMS/ Ag/SiO_2_/Au	Sensing and retaining pressure distribution	≈*µJ*	<4*ms*	Haptic memory	*N/A*	[83]
		PEDOT:PSS/ P(VDF‐TrFE)/ P_3_HT	Tactile sensing and learning	≈*µJ*	≈500*ms*	Handwriting patterns recognition (MNIST)	99.66%	[95]
		ZnO/ PVA@CaCl_2_	Tactile memory	≈*nJ*	≈5*ms*	Image recognition	99%	[96]
	Thermal And electrical	NbO_2_	Voltage and temperature dependent oscillation	*N/A*	*≈ns*	Oscillation neuron	*N/A*	[97]
		PVA/SiO_2_	Temperature dependent synaptic plasticity	≈*pJ*	≈50*ms*	In situ monitoring of environment status	*N/A*	[98]
	Chemical and electrical	TiO_2_ NRs	Gas detecting and learning	≈*µJ*	*≈s*	Infer gas generation location	92.76%	[51]
		PEDOT:PSS	Neurotransmitter‐mediated plasticity	*≈mJ*	*≈s*	Biomedical interfaces	*N/A*	[99]
	Multi‐physical	piezoresistive pressure sensor/NbO_2_	Multisensory fusion and separation	*N/A*	*≈µs*	Pattern recognition and Object classification	100% and 93%	[56]
		Gr/MoS_2_/TENG	Tactile and visual fusion	Self‐powered	≈50*ms*	Handwritten patterns recognition (MNIST)	92%	[100]
		P_3_HT/ PEO NWs	Tactile and visual fusion	*≈nJ*	≈50*ms*	Epidermal gesture recognition	96.3%	[101]
Homogeneous Multimodal Neuromorphic Device	Electrical	Li_x_MoS_2_	Heterosynaptic plasticity	*≈nJ*	≈1*ms*	Synaptic competition and cooperation	*N/A*	[57]
		Ag/AgCl/ Ion‐doped sol–gel silicate/ Si nanowire	Dynamic memory and learning	*≈nJ*	≈100ms	History‐dependent dynamic memory and learning	*N/A*	[102]
		B‐doped n‐Si	Nonlinear mapping	*≈nJ*	*≈ms*	Handwritten patterns recognition (MNIST)	96%	[103]
	Optical	Bi_2_O_2_Se/Gr	Bidirectional All‐Optical Synaptic plasticity	*≈nJ*	≈100*ms*	digital logic functions	*N/A*	[104]
		P_x_O_y_/BP	Fully Light‐Controlled Memory and learning	*≈pJ*	≈10*ms*	Image recognition	96%	[105]

Traditional three‐terminal synaptic transistors utilize gate voltage to modulate channel conductance and employ drain‐source voltage to read the device state.^[^ ^22^, ^106^, ^107^ ^]^ For instance, van de Burgt et al.^[^ ^107^ ^]^ described an electrochemical neuromorphic organic device consisting of an electrolyte that conducts ions but blocks electrons, thereby decoupling the read and write process. More specifically, the gate voltage is applied to control the movement of protons in the electrolyte to regulate the channel conductance, while the voltage applied in the source‐drain region is employed only to read out the conductance change. Differently, Ding et al.^[^ ^108^ ^]^ proposed a WSe_2_‐based memtransistor which utilizes charge trapping/detrapping to achieve simultaneous regulation of channel conductance with gate and drain voltages. Both devices are equipped with three terminals, however, the former is designed with a single terminal to regulate internal state variables, whereas the latter features two regulating terminals, thus earning its designation as a multimodal neuromorphic device.

Similarly, in the case of conventional optoelectronic synapses, both optical and electrical pulses can modulate the channel conductance of the device.^[^ ^29^, ^109^ ^]^ In these devices, only one material is sensitive to light, resulting in a single optical terminal and enabling unidirectional modulation of the device's state variables by light. This typically leads to an increase in synaptic weights, necessitating an electrical signal for decreasing the synaptic weight.^[^ ^110^ ^]^ These devices are considered as heterogeneous multimodal neuromorphic devices due to their regulation by two distinct physical signals. While a novel optoelectronic device consists of multiple materials sensitives to light, thus generating multiple optical terminals, and each of which is sensitive to light with different wavelengths. These optoelectronic devices can realize the bidirectional regulation of synaptic weights under the action of light with different wavelengths to activate corresponding optical terminals and make carriers move in different directions. For example, Li et al.^[^ ^88^ ^]^ demonstrated bidirectional photoresponsive optoelectronic synapses based on In_2_O_3_ (sensitive to ultraviolet with wavelength of 365 nm)/Al_2_O_3_/Y_6_ (sensitive to near‐infrared) phototransistors, thus presenting a homogeneous multimodal neuromorphic device capable of modulation by two identical physical signals.

## 3 Heterogeneous Multimodal Neuromorphic Devices

Heterogeneous multimodal neuromorphic devices can be regulated by a variety of physical signals.^[^ ^111^, ^112^, ^113^, ^114^, ^115^ ^]^ These signals collectively act on internal state variables within the device, and ultimately influence the output properties. As a result, these devices demonstrate excellent abilities of computing in sensor, and can realize unified representation with multiple modalities and spatio‐temporal data pre‐processing. The devices are herein elaborated based on distinct physical signals, including opto‐electric cooperation, mechano‐electric cooperation, thermo‐electric cooperation, chemo‐electric cooperation, and multi‐physical cooperation. The specific working principles of their capacity to perceive and process various physical signals will be carefully delineated.

### 3.1 Opto‐Electric Cooperation for Vision Simulation

The absorption of incident light with a specific wavelength by a semiconductor material results in the generation of electron‐hole pairs,^[^ ^88^, ^110^, ^111^ ^]^ which exhibit distinct properties under diverse external conditions, including the potential for separation to produce a voltage^[^ ^116^, ^117^ ^]^ and temporary residence to alter conductivity.^[^ ^90^, ^118^, ^119^ ^]^ According to the distinct behavior of photogenerated carriers within materials, this section will be divided into four parts for demonstration: classical internal photoelectric effect, photoinduced valance change, photoinduced ferroelectric polarization, and photoinduced phase change. Each section will initially present the physical mechanisms of device modulation by optical signals, followed by an in‐depth exploration of opto‐electric cooperation, displaying the multimodal learning capabilities and their significances for vision simulation.

#### 3.1.1 Classical Internal Photoelectric Effect

The classical internal photoelectric effects, including the photogating effect, carriers captured by cations, and photovoltaic effect based on heterojunctions, represent the most prevalent mechanisms in optoelectronic synapses.^[^ ^116^, ^119^ ^]^ The photogating effect^[^ ^90^, ^91^, ^110^, ^120^, ^121^, ^122^ ^]^ occurs where one type of photogenerated carrier is captured by interface traps, while the other type of photogenerated carrier remains in the channel, causing a change in channel conductance. Carriers captured by cations are associated with anion vacancies in the materials, particularly in amorphous oxide semiconductors (AOS) such as indium‐gallium‐zinc oxide (IGZO),^[^ ^117^, ^123^ ^]^ indium‐zinc oxide (IZO),^[^ ^124^ ^]^ indium‐strontium oxide (ISO),^[^ ^125^ ^]^ and indium oxide.^[^ ^119^ ^]^ This phenomenon leads to the sustained conductivity of these semiconductors even after illumination has been removed. The II‐type heterojunctions^[^ ^126^ ^]^ with barrier structures similar to the PN junctions also enable the effective separation of photogenerated electrons and holes, thereby establishing a sustained build‐in voltage. All of these mechanisms are capable of modulating synaptic weight by light, including synaptic excitation and inhibition, and in some cases, enhancing the sensitivity of devices to light. it is common to use a combination of the aforementioned mechanisms, such as the AOS‐QDs (quantum dots) hetero‐structural active region,^[^ ^117^ ^]^ to maximize the separation of photocarriers and increase their lifetime. However, conventional photoelectric synaptic devices with only one optical terminal can only realize the unidirectional light operation on synaptic weights. They require the use of electrical signals to return the carriers to their initial positions to realize the weights updating operation opposite to the optical signals.

**Figure 3:**
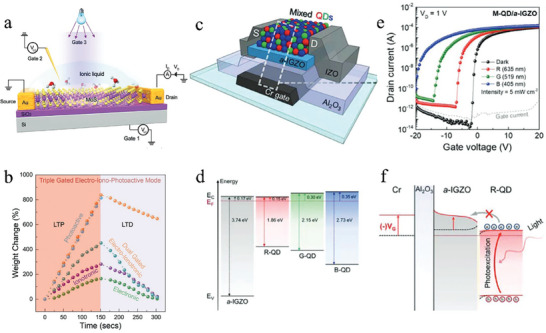
The schematic of multimodal neuromorphic devices based on the classical internal photoelectric effect. a) The proposed multigated architecture of analogous artificial MoS_2_ synapses. b) Controlled facilitation and depression achieved in the device with the multigated architecture. Reproduced with permission.^[^ ^90^ ^]^ Copyright 2018, John Wiley and Sons. c) A schematic structure of the M‐QD/a‐IGZO phototransistor. d) Energy band diagram of R‐, G‐, and B‐QDs and an a‐IGZO with Fermi level alignment. e) Phototransistors under dark and light irradiations, respectively. f) Energy band diagram of R‐QD/a‐IGZO under R‐light irradiation at gate voltage below −8 V. Reproduced with permission.^[^ ^123^ ^]^ Copyright 2022, John Wiley and Sons.

In 2018, John et al.^[^ ^90^ ^]^ reported a MoS_2_‐based neuristor on a silicon substrate (**Figure** **3**a), achieving the modulation of the synaptic weights by optical and electrical signals. The device offers three modulation modes: electronic, ionotronic, and photoactive. When operating in electronic mode, the application of a voltage to the bottom gate leverages the trap state at the semiconductor‐dielectric interface to facilitate hole trapping and de‐trapping. For the ionotronic mode, the migration of ions and carrier concentration in the channel are controlled by applying a voltage across the electrolyte. While operating in photoactive mode, the semiconductor captures photogenerated holes when irradiated by light, leading to an increase in the electron concentration within the channel. These three modulation modes exhibit varying degrees of carrier control, as shown in Figure 3b. Both the electrical and ionotronic mode achieve bidirectional regulation of device conductance (or synaptic weighting), whereas the photoactive mode only achieves an increase in the synaptic weights, and relies on the assistance of the other two modes to achieve the inhibition of the synaptic weights. Simultaneously applying paired stimuli of optical and electrical pulses to the device can mimic classical conditioning and result in associative learning, which facilitates multimodal mapping and co‐learning.

The combination of the three mechanisms mentioned above has led to improve optical response and complex retinal functions. Jo et al.^[^ ^123^ ^]^ developed heterogeneous phototransistors (Figure 3c) for distinct color recognition by constructing a specially RGB mixed QDs (M‐QDs) layer and a semiconducting α‐IGZO layer as the photoabsorber and charge carrying channel, respectively. Figure 3d depicts the energy band diagram of R‐, G‐ and B‐QDs and an α‐IGZO. Multispectral light absorption is achieved by mixing QDs of different sizes with distinct energy bands. When light is incident on the device, a type‐II heterojunction is formed between the two materials, leading to the separation of electrons and holes. This results in the accumulation of holes in the QD layer and migration of electrons to the semiconducting layer. The α‐IGZO layer contains a large number of oxygen vacancies, which can capture electrons and form a persistent photoconductance, thus enabling light‐induced synaptic weight gain. Different kinds of QDs respond to diverse wavelengths of light differently, as shown in Figure 3e, forming three optical terminals akin to cone cells in the retina, enabling them to easily differentiate between the three different wavelengths of light. The modulation of the energy band of α‐IGZO through gate voltage can further affect the optoelectronic response of the device. Specifically, when a negative gate voltage is applied, the conduction band bottom of α‐IGZO is shifted upward as a whole, and when the barrier between α‐IGZO and M‐QDs is larger than the photon energy corresponding to the wavelength of red light, electrons are unable to enter the channel region, resulting in the device being unresponsive to red light (Figure 3f). Based on these findings, the researchers realized full‐range visible color recognition and chromatic control similar to that of the human retina and achieved a 7 × 7 pixel photonic synapse array capable of performing outstanding color image recognition based on adjustable wavelength‐dependent volatility conversion. This device mixes different materials to form multiple optical terminals and uses electrical signals to control the sensitivity of each optical terminal, promising a controlled multimodal fusion strategy.

#### 3.1.2 Photoinduced Valance Change

**Figure 4:**
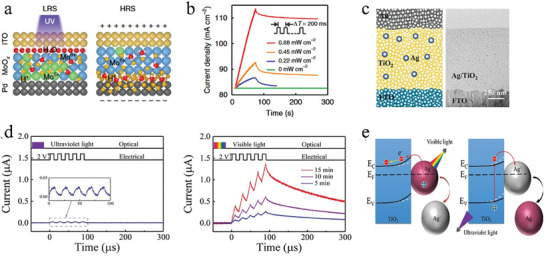
The schematic of multimodal neuromorphic devices based on photoinduced valance change. a) Proposed switching mechanism in the MoOx optoelectronic synaptic device. Mo^5+^ and Mo^6+^ are represented by green and blue balls, respectively. b) Device response through an input light pulse repeated 100 times with a frequency of 2.5 Hz (pulse width, 200 ms; pulse interval, 200 ms) at different light intensities. Reproduced with permission.^[^ ^92^ ^]^ Copyright 2018, Springer Nature. c) Device structure and cross‐sectional TEM image of the Ag–TiO_2_ nanocomposite‐based memristor. d) Current responses under stimulation by pulse strings after UV–visible light irradiation, respectively (2 V, pulse width of 10 µs, interval of 10 µs, read at 0.2 V). e) Schematic diagrams illustrating the mechanisms of light‐induced synaptic modification. Reproduced with permission.^[^ ^127^ ^]^ Copyright 2022, John Wiley and Sons.

Photoinduced valance change^[^ ^92^, ^127^ ^]^ refers to the phenomenon in which photogenerated carriers are either captured by metal ions or metal atoms, leading to a change in the valence state. One scenario involves the capture of photogenerated electrons mental ions, causing an increase in conductivity due to the presence of weakly bound electrons. While under an electric field, the metal ions migrate toward the electrodes to undergo an electrochemical reaction and release the captured electrons, thus returning the device to its initial state. For example, Zhou et al.^[^ ^92^ ^]^ designed a Pd/MoO_x_/ITO optoelectronic synaptic device (**Figure** **4**a) that exhibits light‐ and electric‐tunable synaptic behaviors. Light stimulates the generation of electrons and holes in the ITO layer, with electrons being captured by the oxygen vacancies, while the holes interact with the water molecules adsorbed by the MoO_x_ film to produce protons and electrons.^[^ ^128^, ^129^ ^]^ Catalyzed by the Pd electrode, the protons and electrons lead to the valance change of Mo ions from Mo_6_^+^ to Mo_5_^+^ to form H_y_MoO_x_, accompanied by the transition of resistance states from high resistance state (HRS) to low resistance state (LRS). When a negative voltage is applied to the device, the Mo_5_^+^ ions migrate toward the vicinity of the Pd electrode, leading to an oxidation reaction in which the Mo_5_^+^ ions undergo electron loss and revert back to Mo_6_^+^ ions with reduced conductivity, resulting in a transition of resistance states from LRS to HRS (Figure 4a). As the duration and frequency of the light pulse increase, there is an enhanced likelihood of Mo ion valence changing, facilitating the cumulative increase of synaptic weights and the transition from short‐term synaptic plasticity (STSP) to long‐term synaptic plasticity (LTSP) (Figure 4b). These properties allow the device to perform first‐stage image processing, like image contrast enhancement and noise reduction, efficiently improving the processing efficiencies and accuracy of subsequent processing tasks.

In addition, metal atoms can be excited to generate carriers and subsequently transform into metal ions, a process that can be controlled by an electric field. Shan et al.^[^ ^127^ ^]^ developed a plasmonic optoelectronic memristor (Au/Ag‐TiO_2_/F‐doped SnO_2_, as shown in Figure 4c) that relies on the effects of localized surface plasmon resonance (LSPR)^[^ ^130^, ^131^ ^]^ and optical excitation in an Ag‐TiO_2_ nanocomposite film to realize response to different wavelengths of light and a novel opto‐electric cooperative behavior. When exposed to UV light pulse, TiO_2_ generates electrons that are captured by silver ions, leading to the formation of immobilized Ag atoms in the insulating layer. This process results in a brief spike in current followed by a sustained current below the pre‐lighting level. While exposed to visible light pulses, Ag atoms produce electrons to form Ag^+^ ions, which can be modulated by an electric field, resulting in a transient spike in current followed by sustained current levels above the pre‐lighting baseline. Hence, only after visible light irradiates to the device, the device can only be regulated by electrical signals to realize short‐term potentiation (STP) and long‐term potentiation (LTP) after exposure to visible light, whereas UV irradiation deactivates the device, as shown in Figure 4d,e. This novel opto‐electric cooperative behavior, specifically the light‐gated and electrically‐driven synaptic plasticity, is crucial for pattern recognition in high‐level image processing as it allows for the regulation of synaptic weights in different directions by both optical and electrical signals.^[^ ^132^ ^]^

#### 3.1.3 Photoinduced Ferroelectric Polarization

The process of harnessing spontaneous polarization of the ferroelectric materials to separate photogenerated carriers, which further affect the polarization of the ferroelectric, is referred to as photoinduced ferroelectric polarization.^[^ ^93^, ^133^, ^134^, ^135^ ^]^ Specifically, when light excites the atoms within the ferroelectric, it induces a separation of photogenerated electrons and holes by the built‐in electric field, thus creating an electric field in the opposite direction inside the ferroelectric, and suppressing the ferroelectric polarization.^[^ ^133^ ^]^ In addition, when light excites the atoms around the ferroelectric, such as the channel in the ferroelectric transistor structure, some of the carriers are adsorbed around the ferroelectric, leading to suppression of its polarization.^[^ ^134^ ^]^ Depending on the wavelength of the incident light and the optoelectronic properties of the materials, these two effects can exist separately^[^ ^93^, ^133^, ^135^ ^]^ or simultaneously,^[^ ^134^ ^]^ realizing the modulation of the devices by optical signals. However, the modulation of the ferroelectric polarization by light only exhibits an inhibitory effect, and an electrical signal is required to achieve modulation of the ferroelectric polarization in the opposite direction. For example, Soliman et al.^[^ ^134^ ^]^ investigated the dual electrical and optical regulations of all‐vdW ferroelectric/semiconductor heterostructure. This device combines a CuInP_2_S_6_ (CIPS) ferroelectric layer electrostatically coupled to a ReS_2_ semiconductor channel through a dielectric hBN spacer. Photocarriers are generated in the ReS_2_ layer under 522 nm light irradiation and are adsorbed at the hBN‐ReS_2_ interface under the effect of spontaneous polarization of CIPS, which suppresses the effect of ferroelectric polarization on the channel. In other words, the holes are bound at the interface and the photogenerated electrons remain in the channel, increasing the channel conductance. Under 405 nm light irradiation, ReS_2_ and CIPS are simultaneously excited to produce electron‐hole pairs, while the electron‐hole pairs generated in the ferroelectric form an electric field opposing the polarization direction, thereby partially shielding the ferroelectric's own electric field. Applying a negative voltage pulse to the gate can deplete the photogenerated carriers and restore the ferroelectric polarization to its initial state.

**Figure 5:**
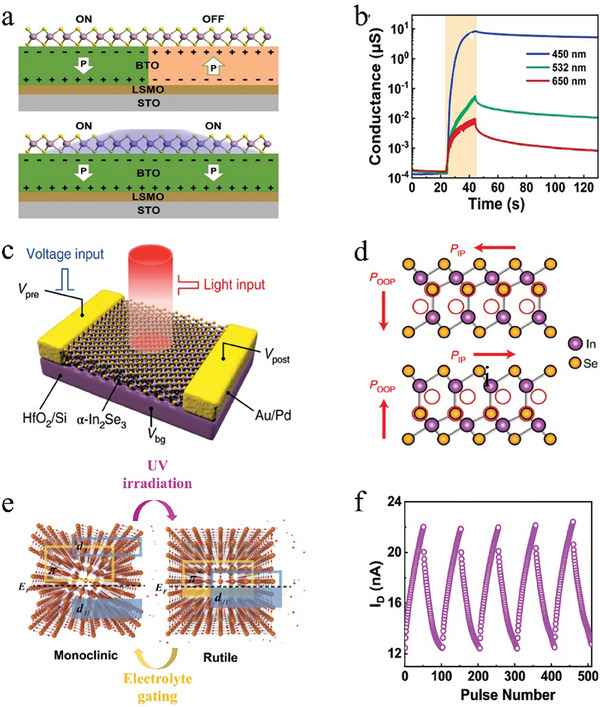
The schematic of multimodal neuromorphic devices based on photoinduced ferroelectric polarization and phase change. a) Schematic diagram of the photo‐induced polarization switching in the BTO film underneath the monolayer MoS_2_. b) The conductance variation as a function of time under different light wavelengths (light intensity of 10 mW cm^−2^, pulse number of 100). Reproduced with permission.^[^ ^135^ ^]^ Copyright 2023, Elsevier. c) Schematic of the optoelectronic synapse with α‐In_2_Se_3_ as the key function material. d) The crystal structure of α‐In_2_Se_3_, depicting the dipole locking effect. The OOP and IP polarizations are induced by the Se atoms in the central layer, which break the centrosymmetry. The simultaneous OOP and IP polarization reversal is induced by the displacement of central Se atoms toward the bottom‐left direction. Reproduced with permission.^[^ ^93^ ^]^ Copyright 2022, Springer Nature. e) Schematic diagram of the reversible non‐volatile phase transition driven by optical and electrolyte gating. f) Light‐controlled LTP (light intensity 84 mW cm^−2^ for 10 s, spaced 10 s apart) and V_G_‐controlled LTD (−1.5 to −3.5 V, duration of 10 s, spaced 10 s apart) for 50 pulses. A constant source‐drain voltage V_SD_ = 50 mV was applied to monitor the channel current. Reproduced with permission.^[^ ^94^ ^]^ Copyright 2022, Springer Nature.

In ferroelectric transistor structures, the photoinduced ferroelectric polarization can be used to modulate the polarization orientation of the ferroelectric gate thus changing the channel conductance to realize the optoelectric synaptic functions. Du et al.^[^ ^135^ ^]^ fabricated the MoS_2_/BaTiO_3_ (BTO) optoelectronic synapse with monolayer MoS_2_ as the light‐sensitive channel and BTO film as the ferroelectric gate. The ferroelectric polarization of devices can be controlled by light and electrical pulses, and thus modulating the device conductance to realize optical excitation and electric inhibition. The light‐controlled ferroelectric polarization switching can be explained in terms of the interaction between the photogenerated charges in MoS_2_ and ferroelectric polarization charges in BTO.^[^ ^136^ ^]^ The asymmetric nature of the MoS_2_/BTO/STO heterojunction results in a preference for the downward polarization state, indicating the presence of the built‐in field oriented toward the bottom electrode. The accumulation of photogenerated positive charge at the MoS_2_/BTO interface screens the upward polarization, which leads to the polarization reversal by the built‐in electric field (**Figure** **5**a). The device exhibits different responses to varying wavelengths of light (450, 532, and 650 nm) as shown in Figure 5b, which is utilized to pre‐process the image data by eliminating the redundant data, and result in an increase in the recognition rate of the MNIST handwritten dataset from 15% to 91% in the simulated neural network. In addition, ferroelectric‐semiconductor junctions can also be optically modulated by ferroelectric polarization, leading to a modification in the device's conductivity. The van der Waals semiconductor α‐In_2_Se_3_ offers ferroelectric,^[^ ^137^, ^138^ ^]^ optoelectronic,^[^ ^139^, ^140^ ^]^ and semiconducting^[^ ^141^, ^142^ ^]^ properties and is potentially an ideal substrate for information processing. Liu et al.^[^ ^93^ ^]^ reported a ferroelectric α‐In_2_Se_3_‐based optoelectronic synapse (Figure 5c) with dynamic temporal responses. The unique asymmetric structure of α‐In_2_Se_3_ allows it to exhibit spontaneous polarization in both lateral and vertical directions (Figure 5d) as well as a dipole‐locking effect,^[^ ^143^, ^144^ ^]^ namely, changing the polarization state in one direction affects the state in the other direction, which makes it possible to simultaneously modulate properties in both the lateral and vertical directions of the device. When a light pulse is applied to the device, the photogenerated carriers change the ferroelectric polarization state of the material in the vertical direction, which in turn affects the lateral ferroelectric polarization state, thus changing the device conductance. Furthermore, the application of an electrical pulse to the device can directly impact the lateral polarization switching and alter the device's conductance. Leveraging the modulation of optical and electrical signals, the researchers developed a reservoir computing system capable of adjustable nonlinear transformations, enabling the fusion of optical and electrical signals.

#### 3.1.4 Photoinduced Phase Change

Phase change is the process in which a material undergoes a structural change from one phase to another.^[^ ^145^, ^146^ ^]^ Photoinduced phase change induced by light irradiation have been demonstrated as the common occurrence,^[^ ^147^, ^148^, ^149^ ^]^ yet there is a scarcity of research applying in electrical neuromorphic devices. Li et al.^[^ ^94^ ^]^ fabricated a VO_2_ optoelectronic synapse that can perceive and memorize UV light stimuli due to its photo‐induced non‐volatile phase change. The activation energy required to create oxygen vacancies falls within the range of 3 to 3.5 eV.^[^ ^150^ ^]^ Therefore, it is anticipated that 375 nm UV light with a photon energy of 3.35 eV, should be capable of releasing oxygen from the VO_2_ film under an oxygen‐deficient environment, thereby generating oxygen vacancies within the crystal lattice. The presence of oxygen vacancies causes V atoms to lose electrons and release them to the neighboring V‐3d states, partially occupying the d_//_ and π^*^ orbitals, leading to an electronic phase transition. At the same time, the appearance of oxygen vacancies induces strain in the structure, causing a structural phase change occurs from monoclinic to rutile and further inducing metallic phases,^[^ ^151^ ^]^ as shown in Figure 5e. The monoclinic phase in the crystals decreases linearly with increasing UV irradiation dose, realizing a light‐regulated linear synaptic weight increase. In contrast, VO_2_ undergoes a transition from a rutile phase to a monoclinic phase under an electric field, resulting in a linear weight decrease (Figure 5f). With its sensitivity to UV light, the optoelectronic synapse array can extract the UV information from the surrounding environment, which significantly improves the image recognition rate on the MNIST handwritten dataset from 24% to 93%. This device can be modulated by both optical and electrical signals, physically expressing the two modalities in a unified way, with excellent sensing and computational capabilities.

### 3.2 Mechano‐Electric Cooperation for Haptic Simulation

When a material is subjected to strain from an external force, it undergoes internal structures and property changes,^[^ ^152^, ^153^, ^154^ ^]^ which can be classified as piezocapacitive effect,^[^ ^155^ ^]^ piezoresistive effect,^[^ ^83^, ^112^, ^156^, ^157^, ^158^, ^159^, ^160^ ^]^ piezoelectric effect,^[^ ^86^, ^95^, ^161^ ^]^ triboelectric effect,^[^ ^162^, ^163^, ^164^, ^165^ ^]^ and other strain‐sensitive effects,^[^ ^96^, ^166^, ^167^ ^]^ depending on the manifestation of the change in material properties induced by the external force. Piezoresistive effects,^[^ ^83^, ^112^, ^157^ ^]^ strain‐induced ferroelectric polarization changed,^[^ ^95^, ^168^ ^]^ and other strain‐sensitive effects^[^ ^96^, ^166^, ^167^ ^]^ have been utilized to realize mechano‐electric cooperation, that is, a mechanical terminal is added while an electrical terminal is retained. This section will be further developed in two parts: the piezoresistive effect and the strain‐sensitive effect, for bio‐plausible Multimodal Learning. The mechanisms of device modulation by mechanical signals, the mechano‐electric cooperation, and the multimodal learning capabilities will be discussed in detail.

#### 3.2.1 Piezoresistive Effect

The piezoresistive effect refers to the phenomenon in which the electrical resistance of a material undergoes changes when subjected to external pressure. Generally, the piezoresistive effect can be enhanced by designing the microstructure of the material to achieve a greater change in resistance under applied stress, for example, by using organic polymers such as polydimethylsiloxane (PDMS)^[^ ^112^, ^158^ ^]^ to fabricate nano‐pyramids arrays on a conductive substrate. Without applied pressure, the tip of the pyramid makes contact with the electrode on the other side, resulting in increased resistance due to the reduced contact area between the two electrodes. Applying pressure to the electrode results in a greater deformation to the sharp tip, leading to an increased contact area between the two electrodes. Moreover, with the increase in pressure, there is a corresponding expansion in the contact area between the two electrodes, leading to a more pronounced alteration in resistance.

A piezoresistive device can be seen as a pressure‐controlled adjustable resistor. Connecting piezoresistive devices and neuromorphic devices in series^[^ ^157^, ^158^ ^]^ allows complex brain‐like computing by modulating the pressure on the piezoresistive device^[^ ^112^ ^]^ as well as the bias voltage of the system^[^ ^157^ ^]^ to alter the output characteristics and responses. Specifically, applying pressure to the piezoresistive device results in a decrease in device resistance, leading to an increase in the voltage divided across the modulating end of the artificial synapse, thereby changing the synaptic weights, which enable both pressure‐ and electrical‐mediated STP and LTP^[^ ^112^ ^]^ as well as the recognition of objects, similar to the human haptic system.^[^ ^156^ ^]^ In addition, the bias voltage can also be applied as an electrical pulse signal,^[^ ^157^ ^]^ in which a constant pressure is exerted on the piezoelectric device as a bias (similar to the role of the bias voltage above), or pressure pulses are applied simultaneously to enable more complex calculations.

**Figure 6:**
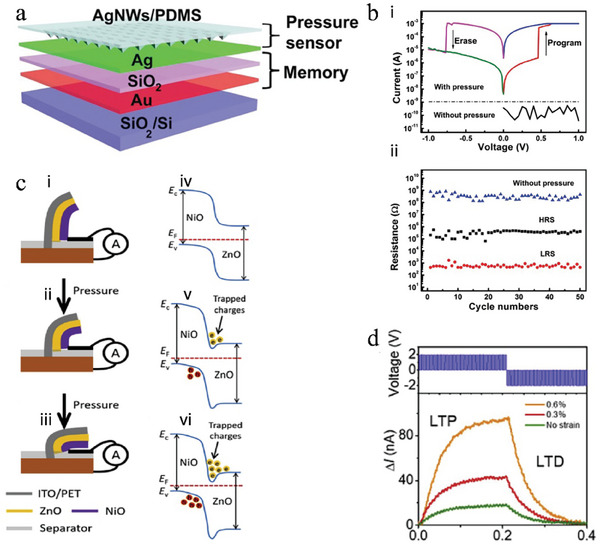
The schematic of multimodal neuromorphic devices based on piezo‐electric cooperation. a) Schematic illustration of a piezo‐electric cooperative device that comprises pressure sensing and resistive switching memory devices. b) Typical current‐voltage curves of the integrated device without and with i) pressure as well as ii) three states in this device. The device can only be programmed and erased when pressure is applied. Reproduced with permission.^[^ ^83^ ^]^ Copyright 2016, John Wiley and Sons. c) Schematic of vertically aligned all‐oxide‐based artificial synapse. Device bending with ii) applied medium and iii) high strains. Band alignment between NiO and ZnO under applied: iv) strain‐free, v) medium, and vi) high strain conditions, indicating the improved change trapping nature. d) Modulation of current by fifty positive and consecutive fifty negative pulses under different applied strains. Reproduced with permission.^[^ ^166^ ^]^ Copyright 2020, Elsevier.

Combining materials with piezoresistive and neuromorphic computational capabilities can constitute a multimodal neuromorphic device that achieves mechano‐electric cooperation. Zhu et al.^[^ ^83^ ^]^ reported a device that mimics human tactile memory by integrating a resistive switching memory device with a piezoresistive sensor (**Figure** **6**a), in which the memory cell can be modulated by an electrical signal after applying pressure to the pressure sensor. Piezoresistive sensors were fabricated utilizing microstructured (micro‐pyramidal) PDMS film embedded with silver nanowires (AgNWs) as the sensitive layer, which gives the pressure sensor high sensitivity at low pressures (<1 kPa).^[^ ^50^ ^]^ The resistive switching memory exploits typical metal‐insulator‐metal (MIM) architecture with SiO_2_ as a switching layer, providing high endurance and nonvolatile memory by virtue of good resistance switching behaviors in SiO_2_.^[^ ^169^ ^]^ Without applied pressure, the piezoresistive sensor demonstrates a high resistance with concentrated voltage drop, resulting in a disordered resistance state within the device. When pressure is applied to the device, the resistance of piezoresistive sensor decreases and the voltage is concentrated in the resistive switching memory, which exhibits a double hysteresis characteristic and can change the resistive state under voltage modulation, as depicted in Figure 6b(i). As a result, the device demonstrates three states (Figure 6b(ii)): a very high‐resistance state without pressure, a high‐resistance state and a low‐resistance state under pressure, allowing the recording of pressure information and the continuous recording of externally applied pressure by electrical modulation. Furthermore, Kim et al.^[^ ^112^ ^]^ utilized the same approach to integrate synaptic transistors and piezoresistive devices. By fixing the voltage at the electrical terminal, and applying pulse pressure across the device, both STP and LTP can be achieved, which facilitates the realization of temporally similar signal processing capabilities found in biology. This result promotes the separation of multimodal information from different objects and the fusion of multimodal information from the same object.

#### 3.2.2 Strain‐Sensitive Effect

Strain‐sensitive effect^[^ ^86^, ^96^, ^166^, ^167^, ^168^ ^]^ involves a significant alteration in the physical properties of the material following the application of strain, thus realizing the modulation of the device through strain. Unlike the use of multiple integrated functional layers to achieve mechano‐electric cooperation as elaborated above, strain‐sensitive materials can accomplish the sensing and processing of strain and other physical signals within a functional layer. For example, the optical modulation function of pressure control can be realized using devices made of materials with strain‐induced piezo‐phototronic effects, such as ZnO.^[^ ^86^ ^]^ The piezo‐phototronic effect results in the generation of oppositely polarized charges at both ends of the material in response to a mechanical stimulus, enabling modulation of carrier generation, transport, separation, and recombination in the optoelectronic device, thereby realizing a multistage synaptic response. Mechanical deformation can also alter the ion transport characteristics of organic solid dielectrics,^[^ ^170^ ^]^ leading to strain sensitivity of organic electrochemical transistors made with them. Strain influences the decay constant of the transistor, potentially facilitating a pressure‐induced, electrically modulated transition from STSP to LTSP. In addition, Strain can change the spontaneous polarization of ferroelectrics like electrical signals, thereby impacting their electrical properties. Lee et al.^[^ ^95^ ^]^ fabricated a ferroelectric‐gate field‐effect transistor with a dome‐shape tactile top‐gate, the polarization state of the ferroelectric layer can be altered by applying pressure on the device in addition to the regulation of ferroelectric polarization state by electrical signals, realizing strain‐electric coordination by using the modulation effect of pressure and electricity on ferroelectric switching.

Changing the configuration of the material stack can improve the sensitivity of the device to strain, realizing more significant mechano‐electric cooperation. In 2020, Kumar et al.^[^ ^166^ ^]^ developed a simple two‐terminal flexible piezotronic artificial synapse that mimics environment‐adaptable tactile perception. The device consists of a ZnO piezoelectric layer and a NiO charge‐blocking layer, which form a straightforward heterojunction with electrical synaptic functionality. Furthermore, the device is vertically aligned to enhance deformation in response to external forces. As the strain increases, the bands align in a manner that allows for a greater trapping of charges at the interface (Figure 6c). This results in electrical pulses with the same parameters but varying abilities to modulate the device weights at different degrees of strain, indicating an improvement in modulation efficiency as the degree of strain increases, as in Figure 6d. The simple design and high sensitivity of these devices can be utilized to create efficient human‐like tactile sensing systems, and integrating them into arrays further enables spatio‐temporal processing capabilities for external strains (i.e., strain amplitude and duration). Chen et al.^[^ ^167^ ^]^ similarly implemented human‐like tactile sensing and gesture recognition using a MoTe_2_‐based memristor, successfully extracting tactile information by taking advantage of the device's more pronounced response to the same electrical pulse under strain, facilitating the extraction and integration of multimodal data within the environment.

### 3.3 Thermo‐Electric Cooperation for Thermoreception Simulation

Temperature is an indication of the average kinetic energy of molecular motion within an object. As the temperature increases, the thermal motion of molecules intensifies, potentially leading to phase changes within the material structure,^[^ ^56^, ^97^, ^171^, ^172^ ^]^ thermal activation of molecules to produce free carriers and unoccupied states^[^ ^84^, ^173^ ^]^ as well as an increase in the diffusion rate, thus achieving thermal equilibrium easily.^[^ ^98^, ^113^, ^173^ ^]^ The temperature‐dependent electrical modulation processes of various conventional neuromorphic devices, including Mott memristors, synaptic transistors based on carrier trapping/de‐trapping, and ion‐gated synaptic transistors, will be specified to illustrate their multimodal learning capabilities through thermo‐electric cooperation.

**Figure 7:**
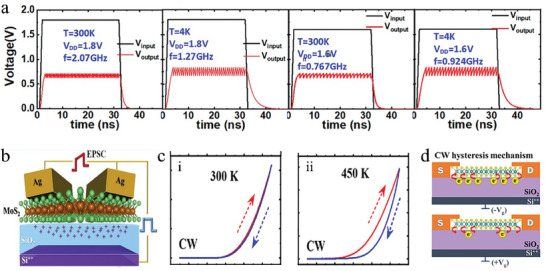
The schematic of multimodal neuromorphic devices based on thermo‐electric cooperation. a) Simulated oscillation waveforms with various V_DD_ and temperature values. Reproduced with permission.^[^ ^97^ ^]^ Copyright 2020, American Institute of Physics. b) The structure schematic of MoS_2_/SiO_2_/Si three‐terminal devices. c) Dual sweep hysteresis behaviors on CVD‐grown MoS_2_. d) Charge‐(de)trapping processes in clockwise hysteresis at high negative and positive gate voltages. Reproduced with permission.^[^ ^84^ ^]^ Copyright 2023, American Chemical Society.

The Mott memristor is a device exhibiting threshold switching (TS) characteristics, which mimics synaptic STP and neuronal behaviors.^[^ ^174^, ^175^ ^]^ When applying a constant voltage across the Mott memristors, such as NbO_x_,^[^ ^52^ ^]^ Joule heat is generated within the device, and simultaneous heat exchange with the external environment occurs, ultimately leading to a stable internal temperature equilibrium. When the voltage surpasses the phase change threshold, the temperature within the device exceeds the material's phase transition temperature, resulting in a reversible transition from insulating to metallic phase, and a decrease in resistance. When the constant voltage is removed, the temperature inside the device gradually decreases to the ambient temperature through heat exchange, causing the reverse phase transition and restoration of the device's resistance. By connecting a Mott memristor with a capacitor and resistor, it is possible to achieve a spiking neuron.^[^ ^56^, ^97^ ^]^ Careful design of the series resistance and DC bias can make the load line of the resistor cross the hysteresis window. When the device is operated in this regime, the voltage across the Mott memristor in HRS exceeds the upper threshold while the voltage across the LRS falls below the lower threshold, resulting in a continuous oscillation waveform, which creates a neuron with a firing function. The spike amplitude and the firing rate of the neuron are capable to be adjusted through the magnitude of the voltage and the initial temperature of the device or external temperature (**Figure** **7**a). As a result, the electrical and temperature signals can be extracted based on the spike amplitude and rate.^[^ ^56^ ^]^

Synaptic transistors that rely on carrier trapping/de‐trapping can utilize electric field modulation to simulate synaptic function.^[^ ^173^ ^]^ However, at low temperatures, carriers are trapped in deep energy level traps, resulting in a weak modulation effect of the electric field and preventing the material from exhibiting carrier trapping effects. As the temperature rises, the Fermi level approaches the deep energy level, resulting that these traps release carriers and form an empty trap state. This allows for the process of carrier trapping and de‐trapping under an electric field. The interfacial states at the dielectric‐semiconductor interfaces are also gradually excited as the temperature rises, releasing carriers into the channels and enhancing the efficiency of the electric field in regulating the device. Mallik et al.^[^ ^84^ ^]^ have demonstrated a MoS_2_/SiO_2_/Si three‐terminal device (Figure 7b) with distinct functionalities that vary with temperature changes, such as high‐gain intrinsic transistors operated at room temperature, and synapse‐like multilevel storage functions exhibited above room temperature, as shown in Figure 7c. At ambient temperature, the trap states of the MoS_2_‐dielectric interface remain inactive, causing good hysteresis‐free characteristics. Above the threshold temperature of 450 K, the deep energy levels at the interface are activated,^[^ ^176^ ^]^ and an electric field‐driven trapping/de‐trapping process of carriers is realized, resulting in hysteresis and synapse‐like multilevel memory, as shown in Figure 7d.

Ion‐gated synaptic transistors achieve modulation of the channel conductance by controlling the migration of ions in the electrolyte under an electric field and the Coulombic effect of ions on channel carriers, thereby realizing STP and LTP.^[^ ^22^ ^]^ Within a certain temperature range, the mobility and diffusion coefficient of ions increase, resulting in more ions migrating to the electrolyte‐channel interface under the same electric pulse. Additionally, the ions are more loosely distributed in the electrolyte during a long duration electrical pulse, leading to a decrease in the amplitude and duration of the postsynaptic current. Zhu et al.^[^ ^113^ ^]^ prepared an ITO/chitosan/IGZO ion‐gated transistor by exploiting temperature modulation of the ions and a brief electrical pulse to induce an increase in postsynaptic current due to temperature elevation. In contrast, Fu et al.^[^ ^173^ ^]^ investigated the temperature dependence of IGZO electrochemical gating transistors with sputtered SiO_2_ electrolytes, in which two temperature modulation mechanisms are present: thermal activation of the interface state and temperature‐dependent ion mobility in electrolytes. As the temperature increases, the interface state releases carriers into the channel, thereby augmenting the initial postsynaptic current of the device. A prolonged electrical pulse, however, has a reduced modulating effect on the device, leading to a quicker decrease in the increment of postsynaptic current and a return to the initial state. These two studies demonstrate the modulating effect of temperature on the device and reveal the potential of utilizing thermo‐electric cooperation to achieve a more bio‐plausible electronic skin with multimodal learning capabilities.

### 3.4 Chemo‐Electric Cooperation for Smell and Taste Simulation

The interaction of materials with inorganic substances including different gas molecules^[^ ^177^ ^]^ and water molecules,^[^ ^178^ ^]^ can be combined with neuromorphic devices to mimic biological olfactory^[^ ^51^ ^]^ and humidity sensing^[^ ^85^ ^]^, thereby building multimodal systems more in line with biological behavior. While the material's response to organic matter, especially certain neurotransmitters in living organisms,^[^ ^99^ ^]^ is conducive for researchers to build brain‐computer interfaces with multimodality‐like brains and realize communication and collaboration between biological systems and electronic systems.

**Figure 8:**
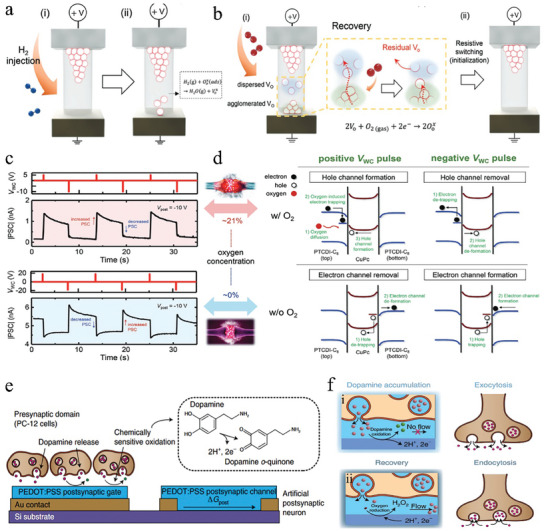
The schematic of multimodal neuromorphic devices based on chemo‐electric cooperation. a) Schematic illustration of the sensor response to H_2_ in the initial state (i) and after the generation of oxygen vacancies (ii). b) Schematic illustration of the recovery process by oxidation and sporadic distribution of residual oxygen vacancies (i) and manual initialization of residual oxygen vacancies through resistive switching (ii). Reproduced with permission.^[^ ^51^ ^]^ Copyright 2023, John Wiley and Sons. c) Schematics of corresponding energy band diagrams and charge trapping behavior of trilayered organic double heterojunction in the four cases in (d). d) PSC responses under the application of positive and negative weight‐control voltage pulses at oxygen concentrations of 21% (top) and 0% (bottom). Reproduced with permission.^[^ ^180^ ^]^ Copyright 2020, John Wiley and Sons. e) The oxidation of dopamine (pink spheres) to dopamine o‐quinone (green spheres) at the postsynaptic gate electrode controls the change in conductance of the postsynaptic channel. f) Accumulation of dopamine at the cell–PEDOT: PSS interface occurs under a low flow rate (i) and accumulation of dopamine at the cell–PEDOT: PSS interface occurs under a high flow rate (ii). Reproduced with permission.^[^ ^99^ ^]^ Copyright 2020, Springer Nature.

The perception of gas molecules, including NO_2_,^[^ ^114^, ^179^ ^]^ H_2_,^[^ ^51^ ^]^ NO,^[^ ^51^ ^]^ O_2_,^[^ ^180^ ^]^ H_2_S,^[^ ^177^ ^]^ etc., by neuromorphic devices is realized through redox reactions. These redox reactions manifest as the depletion or generation of channel carriers, the generation or depletion of oxygen vacancies in insulators, the induction of defective states in the channel, and the enhancement of ionic mobility within the electrolyte. In the study of Yuan et al.,^[^ ^179^ ^]^ NO_2_ undergoes oxidation in the α‐IGZO N‐type semiconductor channel, leading to carrier depletion and generation of NO_2_^−^ ions, which subsequently reduce the electron mobility in the channel, realizing varying spike firing rates at different NO_2_ concentrations. While in the work of Song et al.,^[^ ^114^ ^]^ the oxidation reaction of NO_2_ in the P‐type semiconductor channel generates additional holes, also realizing the gas pulse‐induced postsynaptic currents equivalent to those induced by the electric pulses. Chun et al.^[^ ^51^ ^]^ also fabricated a Pt/TiO_2_ nanorods (NRs)/TiN‐structured device to achieve gas‐pulse‐induced plasticity. When the device is exposed to 1% H_2_ at room temperature (25 °C), the hydrogen molecules come into contact with the surface of the metal oxide NRs, which undergo a reduction reaction to form oxygen vacancies and release the electrons trapped by the surface oxygen.^[^ ^181^ ^]^ thereby increasing the conductance of the device as shown in **Figure** **8**a. Whereas the passage of oxidizing gases, such as NO or O_2_, to the device leads to oxidation and subsequent filling of oxygen vacancies in the device, resulting in a decrease in the device conductance as shown in Figure 8b. While Qian et al.^[^ ^180^ ^]^ realized another form of synergy between oxygen and electrical signals by employing an oxygen‐sensitive organic double heterojunction with PTCDI‐C8/CuPc/PTCDI‐C8 structure. They utilized the oxygen‐induced electron trapping in PTCDI‐C8 to modulate majority charge from holes to electrons, and induced an interconversion between excitation and inhibition modes depending on the oxygen conditions in the environment. In the absence of oxygen in the surroundings, a large number of hole traps are present in CuPc. As the concentration of oxygen increases, the oxygen‐induced electron traps in the organic layer increase, which consequently neutralizes the hole traps and dominates the modulation of the device progressively (Figure 8c). As a result, an increase in the postsynaptic current is observed when a negative voltage is applied at a low oxygen concentration with majority carrier electrons, whereas the opposite effect is seen at high oxygen concentration (Figure 8d). By bonding to a breath‐figure derived porous solid polymer electrolyte (SPE), Deng et al.^[^ ^177^ ^]^ developed an organic electrochemical transistor with PEDOT: PSS as the channel. When H_2_S gas is introduced, it interacts with the SPE and establishes ionic contacts with [BMIM] [TFSI] in SPE, thereby weakening the electrostatic interactions between [BMIM]^+^ cations and [TFSI]^−^anions, and promoting cluster formation and ion mobility. This results in easier movement of ions^[^ ^182^ ^]^ and realize the transition of the device from STP to LTP.

Neuromorphic devices exhibit a similar response to organics, which is achieved through redox reactions. A notable instance is the synergistic dopamine and electrical modulation achieved by Keene et al.^[^ ^99^ ^]^ using PEDOT: PSS organic electrochemical transistors. In their proposed biological hybrid synapse, dopamine released by PC‐12 cells at the presynaptic tip was locally oxidized at the postsynaptic gate electrode (Figure 8e), and a microfluidic channel fabricated by PDMS was used to facilitate the recycling of dopamine and its oxidation product at the artificial synapse. At low flow rates (Figure 8f(i)), dopamine accumulates at the cell‐PEDOT: PSS interface, and subsequent oxidation of dopamine at the postsynaptic gate electrode surface lead to memory conditioning. At high flow rates (Figure 8f(ii)), dopamine is removed from the cell‐PEDOT: PSS interface, followed by reduction, leading to memory recovery and mimicking endocytosis. This paves the way for the integration of artificial neuromorphic systems with biological neural networks using multimodal neuromorphic devices.

### 3.5 Multiple Physical Cooperation for Multisensory Perception

**Figure 9:**
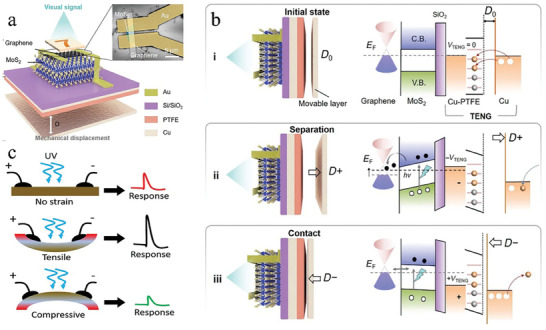
The schematic of multimodal neuromorphic devices based on multi‐physical cooperation. a) Schematic diagram of the mechano‐photonic artificial synapse based on graphene/MoS_2_ (Gr/MoS_2_) heterostructure. b) Working mechanism of the mechano‐optoelectronic transistor based on Gr/MoS_2_ heterostructure. Schematic illustrations of the working principles and the corresponding energy band diagram at i) initial flat‐band state, ii) separation state (D+), and iii) contact state (D−). Reproduced with permission.^[^ ^100^ ^]^ Copyright 2021, Science. c） The optical pulses work as an action potential, while measured photocurrent is a synaptic response. Modulation of the photocurrent generation with no strain, tensile strain, and compressive strain, respectively. Reproduced with permission.^[^ ^86^ ^]^ Copyright 2019, Elsevier.

This section discusses neuromorphic devices capable of sensing multiple distinct physical signals, such as combinations of electrical, optical, mechanical, thermal, and chemical stimuli. One method of achieving tight integration to realize multi‐physical cooperative devices is by replacing one of the electrical terminals of a multiterminal device with other physical terminals. Mechano‐optic cooperative devices^[^ ^55^, ^100^, ^183^, ^184^, ^185^ ^]^ have been developed by substituting the gate electrodes of opto‐electric cooperative devices with a piezoelectric or triboelectric nanogenerator (TENG) material that can transform the pressure signal into an electrical signal. For instance, Yu et al.^[^ ^100^ ^]^ fabricated a mechano‐photonic artificial synapse with synergistic mechanical and optical plasticity by integrating graphene(Gr)/MoS_2_ heterojunction‐based phototransistor with a TENG (**Figure** **9**a). The application of an external force results in a modification of the spacing of TENG, leading to the generation of either a negative or positive polarization electric field. The negative polarization (Figure 9b(ii)) enhances the separation of photogenerated carriers, which then migrate into graphene and recombine with holes, causing a reduction in channel conductance and retention for a period under interfacial barriers.^[^ ^186^ ^]^ The positive polarization (Figure 9b(iii)) corresponds to applying a positive gate voltage to the device, which promotes the redistribution of carriers in the heterojunction and realizes the erasure process. The artificial neural network constructed using this bimodal device achieves higher accuracy in image recognition tasks. In addition, in the spiking neuron configuration, replacing the resistor in series with the Mott memristor with a varistor modulated by other physical signals, such as a piezoresistive device, allows for the synergistic modulation of thermal and other physical signals. Zhu et al.^[^ ^56^ ^]^ connected a pressure sensor based on piezoresistive effect in series with a NbO_x_ Mott memristor. By utilizing the temperature to change the threshold voltage of the NbO_x_^[^ ^97^ ^]^ and the pressure^[^ ^156^ ^]^ to alter the partial voltage of the memristor, they successfully integrated two different physical signals into the form of electrical signals, and separated the two physical signals with the two parameters of frequency and amplitude, thereby realizing the multimodal integration^[^ ^38^ ^]^ and separation^[^ ^35^ ^]^ of the biological brain.

Another approach to develop multi‐physical cooperation involves the integration of diverse physical mechanisms within a functional material layer, utilizing the varied physical properties of the material to realize the perception of different physical signals. For example, some flexible materials^[^ ^101^ ^]^ exhibit excellent optoelectronic properties and can be modulated by external forces in addition to being regulated by light, as they undergo strain to alter these properties in response to external stimuli. Kumar et al.^[^ ^86^ ^]^ reported a flexible two‐terminal ZnO/AgNWs/PET photonic synapse that utilizes the piezo‐phototronic properties induced by ZnO strain to achieve vastly different light signal modulation under varying strains. When the device is under tensile strain, its optical response is enhanced compared to its response to UV light when no strain occurs. Conversely, the optical response is weakened when the device is under compressive strain, as shown in Figure 9c. Jiang et al.^[^ ^187^ ^]^ also used the ZnO with piezoelectric properties and MoO_x_ to establish a heterojunction to achieve pressure‐induced and light‐induced synaptic enhancement, as well as electrically‐induced synaptic inhibition. This result suggests that α‐In_2_Se_3_, which possesses with both ferroelectric^[^ ^137^ ^]^ and optoelectric^[^ ^139^ ^]^ properties, may hold promise for the realization of mechano‐photonic cooperation with simple structures. Analogous to the above elaboration, the effect of temperature on the device can also be integrated into optoelectronic synergistic devices. Chen et al.^[^ ^188^ ^]^ constructed a temperature‐dependent memory/synaptic hybrid artificial neuromorphic device based on a floating‐gate transistor using CsPbBr_3_/TiO_2_ core‐shell nanocrystals as the photosensitive layer. As the temperature increases, the activation energy of the captured electrons decreases, increasing the probability of electron de‐trapping and recombination with holes. This leads to a gradual transition from non‐volatile to volatile modulation of the device by light and electricity. In addition, in some cases, the chemo‐electric cooperative device can also integrate the optical regulating function.^[^ ^115^ ^]^ Wang et al.^[^ ^85^ ^]^ designed a multimodal memristor containing MXene nanosheets/ZnO nanoparticle heterostructure, which can separate the photogenerated carriers and adsorb water molecules to restrict the growth of the oxygen vacancies conductive filaments, so that realizing a mixture of visual data sensing, RH sensing, and pre‐processing functions. The above findings demonstrate the potential for discovering additional mechanisms in materials to realize multifunctional and multisensory devices with straightforward structures.

## 4 Homogeneous Multimodal Neuromorphic Device

This section will focus on the homogeneous multimodal neuromorphic devices which can be simultaneously modulated by multiple same‐physical signals (i.e., multiple electrical signals or multiple optical signals).^[^ ^87^, ^88^, ^189^, ^190^, ^191^, ^192^, ^193^ ^]^ The homogeneous multimodal neuromorphic devices demonstrate powerful regulation and rich functionality, so as to enable complex brain‐like computations those are difficult to be achieved using two‐terminal in‐memory devices. The homogeneous multimodal devices are equipped with electronics and optics tailored to common signal types, showcasing their exceptional computational capabilities.

### 4.1 Electronics for Building Complex Networks

Adding extra electrical terminals to the two‐terminal in‐memory device structure is a technique for creating homogeneous multimodal neuromorphic devices, as the mechanisms closely resemble those found in in‐memory devices, including ion migration,^[^ ^127^, ^194^, ^195^ ^]^ carrier migration,^[^ ^196^, ^197^, ^198^ ^]^ phase transition,^[^ ^57^ ^]^ and Schottky barrier modulation.^[^ ^189^ ^]^ However, homogeneous multimodal neuromorphic devices prioritize the dynamical processes inherent in each mechanism, with each modulation terminal capable of influencing the entire material layer and enabling signal coupling from multiple modulation terminals. Depending on the different functions of the device, this section is divided into three parts: heterosynaptic plasticity, neuronal nonlinearity, and higher‐dimensional functionality. Each part corresponds to the device's simulation of different units within the central nervous system that are crucial for the brain's multimodal learning, and gradually delineates the intricate computational capabilities thus achieved.

#### 4.1.1 Heterosynaptic Plasticity

Most studies have primarily focused on the assumption that synapses are solely controlled by pre‐ and post‐neurons, while in reality biological synapses can be modulated by multiple neurons to change their properties.^[^ ^22^, ^199^, ^200^, ^201^, ^202^ ^]^ In the case of heterosynaptic plasticity, synapses can also be controlled by other interneurons in addition to pre‐ and post‐neurons,^[^ ^203^, ^204^, ^205^ ^]^ which contributes to a variety of neural processes, including associative learning, neural circuit development, and homeostasis of synaptic inputs. Homogeneous multimodal neuromorphic devices have multiple modulation terminals,^[^ ^194^ ^]^ and the signals between the terminals can be coupled to each other,^[^ ^196^ ^]^ which allows the simulation of heterosynaptic plasticity with a single device for multiple neural processes.

**Figure 10:**
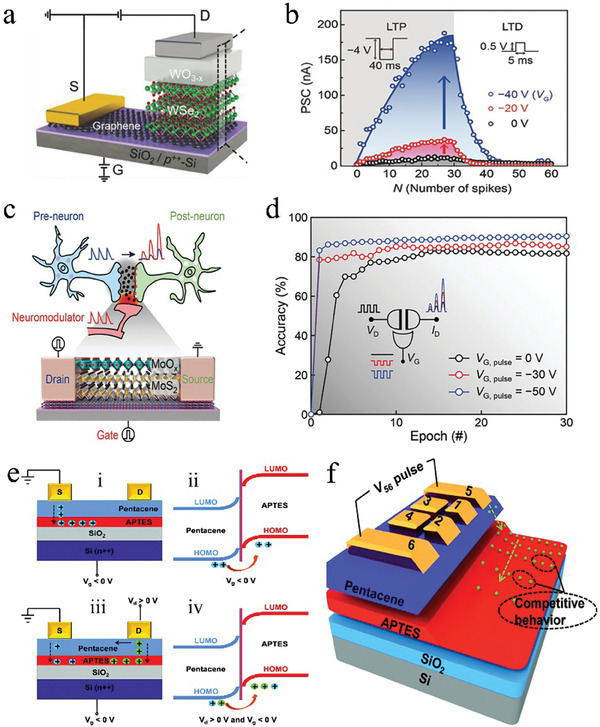
The schematic of heterosynaptic plasticity using homogeneous multimodal devices in electrics. a) Circuit and schematic representation of the synapse consisting of a vertically integrated WO3‐x memristor and WSe2/graphene tunable junction. b) Plots of PSC as a function of the number of input spikes while consecutively applying a series of potentiating spikes (V_A_ = −4 V, V_W_ = 40 ms, and N = 30) and depressing spikes (V_A_ = 0.5 V, V_W_ = 5 ms, and N = 30) at various V_G_ values of 0, −20, and −40 V. Reproduced with permission.^[^ ^206^ ^]^ Copyright 2018, John Wiley and Sons. c) Schematic of biological neurons (top) and a heterosynaptic artificial synapse comprising surface‐oxidized MoS_2_ on the h‐BN/SiO_2_ (285 nm)/Si substrate, presenting drain, source, and gate terminals acting as a pre‐neuron, post‐neuron, and modulator, respectively (bottom). d) Recognition accuracy for MNIST pattern recognition as a function of training epochs for different V_G_, pulse values (−50, −30, and 0 V). Reproduced with permission.^[^ ^207^ ^]^ Copyright 2023, John Wiley and Sons. e) Schematic illustration of the memtransistor operation (i, iii) and energy band diagram (ii, iv). f) Schematic illustration of the multi‐terminal memtransistor, which is analogous to the synaptic competitive behavior. Reproduced with permission.^[^ ^211^ ^]^ Copyright 2020, Royal Society of Chemistry.

The extra modulation terminals increase the tunability of the devices, allowing them to facilitate bidirectional changes in weights but also increase the range and stability of synaptic weight, thereby further improving the overall performance of the neural network. In 2018, Huh et al.^[^ ^206^ ^]^ demonstrated the effect of additional tuning terminals on synaptic weight range and plasticity by utilizing a three‐terminal artificial synapse consisting of a vertically integrated monolayer WO_3‐x_ memristor and a barrier‐tunable WSe_2_/graphene Schottky diode (**Figure** **10**a). With the application of voltage pulses solely at the drain, there is a progressive accumulation of oxygen vacancy density at the WO_3‐x_/Ag interface with an increasing number of pulses, leading to a modification of the device's resistance. When a negative voltage is applied to the gate, the Schottky barrier of the WSe_2_/graphene junction decreases, and the effective electric field across the WO_3‐x_ layer is enhanced, leading to enhanced modulation capability for pulses, as well as an increase in the dynamic range of the PSC. As the negative gate voltage increases, the device achieves a transition from STSP to LTSP, and consolidates LTSP, as shown in Figure 10b. Similarly, in 2023 Huh et al.^[^ ^207^ ^]^ realized the modulation of synaptic weight dynamic range and resistance ratio by an additional tuning terminal, using a three‐terminal heterosynaptic memory transistor with intentional‐defect‐generated MoS_2_ channels (Figure 10c), in which an electric field can control the movement of the defects in the channel. By applying a negative gate voltage pulse, the Fermi level of MoS_2_ is pulled down toward the edge of the valence band, forming more trap states and enhancing the modulation of the device by the source‐drain voltage pulse. This, in turn, enhances both the dynamic range of the device resistance as well as the resistance ratio. A neural network was also built, and as the negative voltage pulse at the gate increased from 0 to −50 V, training for 30 epochs resulted in an accuracy improvement from 81% to 91%, with the number of epochs required to achieve the same accuracy (80%) decreasing from 12 to 1, as in Figure 10d, significantly improving the performance of the neural network.

Signals from various modulation terminals can be interconnected, thereby simulating synaptic interactions, including competition and cooperation.^[^ ^208^, ^209^, ^210^ ^]^ These behaviors may contribute to the development of distinct brain regions and support the integration and transition between different brain regions, thereby facilitating multimodal learning.^[^ ^57^, ^211^ ^]^ In 2018, Sangwan et al.^[^ ^189^ ^]^ fabricated a multiterminal hybrid memtransistor using polycrystalline monolayer MoS_2_, which can simulate synaptic interactions. Applying a voltage to any terminal creates a distributed potential within the device, leading to the migration of defects in the material. This results in a modification of the Schottky barrier distribution and consequently alters the equivalent resistance between any two terminals. Moreover, this six‐terminal structure enables plasticity between any two terminals and their tuning by other terminals, promising more complex learning from multiple inputs that mimic biological neurons with multiple synapses. To take it further, Zheng et al.^[^ ^211^ ^]^ fabricated a three‐terminal memtransistor with an organic self‐assembled layer of 3‐aminopropyltriethoxysilane (APTES), realizing synaptic competition and cooperation. During the self‐assembly process, hydrogen bonding between APTES molecules leads to the formation of defects in the aggregates at the interface,^[^ ^212^, ^213^ ^]^ thus the device can utilize both gate and drain capture of carriers to change the carrier concentration in the channel. By leveraging the superposition property of voltage, the simultaneous application of positive and negative voltages at the drain and gate, respectively, can drive the migration and capture of holes in the channel more efficiently, thus realizing the cooperation between synapses, as shown in Figure 10e. In addition, due to the distribution property of the electric field, the electric field strength is higher near the applied voltage end, making hole migration and capture easier and realizing synaptic competition, as shown in Figure 10f. It may provide novel bioinspired ideas for fusing multimodal information occurring in different times and spaces.

#### 4.1.2 Neuronal Nonlinearity

In contrast to traditional devices that utilize multiple components to simulate single‐input and single‐output neurons,^[^ ^10^, ^214^, ^215^, ^216^, ^217^ ^]^ biological neurons are more compact, featuring multiple dendrites and an axon with numerous branches, which provide more input and output terminals. This configuration forms the brain's intricate 3D network of connections, and endows the brain with superior spatio‐temporal information processing capabilities.^[^ ^218^, ^219^, ^220^ ^]^ In addition, the functionality of neurons in the brain is not singularly fixed, but rather varies according to the type of task. Specifically, the overlapping parts of different brain regions change their function and type as data flows,^[^ ^34^, ^35^ ^]^ contributing to the brain's multimodal learning capabilities. These biological neuronal functions mentioned above including complex connectivity,^[^ ^87^ ^]^ spatio‐temporal information processing,^[^ ^192^, ^198^ ^]^ and functionality changing with processing data^[^ ^102^, ^221^ ^]^ are all nonlinear and can be realized using homogeneous multimodal neuromorphic devices with more terminals.

**Figure 11:**
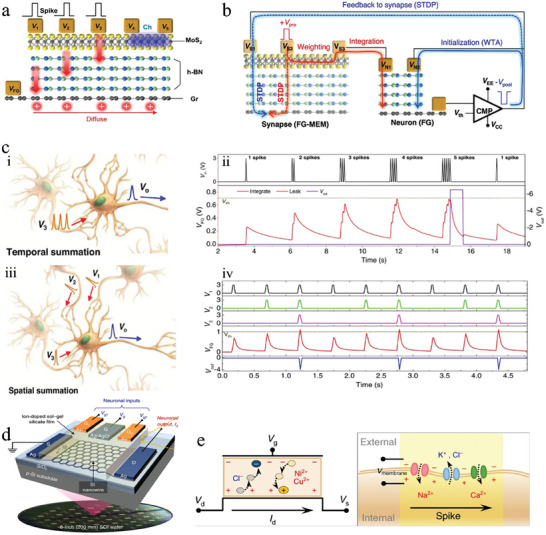
The schematic of neuronal nonlinearity using homogeneous multimodal devices in electrics. a) Cross‐sectional schematics, and operation principle of multi‐terminal floating‐gate memristor. b) Schematics of basic synapse‐neuron assembly for unsupervised learning process by synaptic STDP and neuronal LIF functions. c) Schematics of (i, ii) temporal and (iii, iv) spatial summation in biological neurons. Reproduced with permission.^[^ ^87^ ^]^ Copyright 2023, Springer Nature. d) Schematic diagram of scalable neurotransistors with multiple neuronal input gates V_gi_ and I_d_. e) Illustration of the structural similarity between the ion migration in the neurotransistor (up) and the membrane of a neuron cell in which the ionic current was modulated by a membrane potential (V_memb_) change in the case of the action potential (down). Reproduced with permission.^[^ ^102^ ^]^ Copyright 2020, Springer Nature.

In 2023, Won et al.^[^ ^87^ ^]^ implemented multi‐neuron connection using a multiterminal floating gate transistor with a graphene/insulator/metal heterostructure (**Figure** **11**a) and successfully realized the spatio‐temporal information processing capability of neurons. The device utilized the property of variable Fermi levels of graphene^[^ ^222^, ^223^ ^]^ to obtain a larger memory window and allowed charging and discharging of the floating gate using horizontally distant multiple electrodes. With a negative voltage applied on any electrode V_1_‐V_5_, electrons tunnel through the hBN and accumulate in the graphene layer, which leads to a negative voltage on the floating gate and causes the energy band of MoS_2_ to bend upward, thus decreasing the channel conductance. While applying a positive voltage, tunneling holes accumulate in the graphene layer, resulting in a positive voltage and bends the MoS_2_ band downward, increasing the channel conductance. Without applying voltage, the electrons/holes revert back to the MoS_2_ layer, returning the device to its initial state. This process successfully simulated the change in neuronal membrane potential and synaptic weight alteration under external stimuli. Connecting this device with a comparator (Figure 11b) enables the leaky integrated firing function and spatio‐temporal information processing capability of the neuron (Figure 11c), which is conducive to the integration and separation of the signals of multiple modalities.

The variation in functionality as a result of neuronal data processing may be attributed to intrinsic plasticity. Baek et al.^[^ ^102^ ^]^ reported a neurotransistor made of silicon nanowire transistors coated with an ion‐doped silicate sol‐gel film that mimics the intrinsic plasticity of neuronal membrane (Figure 11d). Driven by the gate voltage higher than the ionic barrier, the ions in the sol‐gel‐derived film experience a directional movement, which is rearranged in the film to form a space charge polarization, resulting in the output characteristics of the device being affected by the input history, as shown in Figure 11e. This device has multiple input and output terminals, and can convert multiple input signals through nonlinear processing via sigmoidal transformation into multiple output currents. The output dynamic can control the time of reaching spike thresholds and the rate of spike, enabling dynamic learning capabilities, which are expected to enable multimodal integration and co‐learning in a manner more consistent with biological behavior. In addition, Beck et al.^[^ ^221^ ^]^ demonstrated electrostatic control of dual‐gated Gaussian heterojunction transistors (GHeTs) for simplified spiking neuron implementation, which employed wafer‐scale MoS_2_/single‐walled carbon nanotubes mixed‐dimensional heterojunctions to emulate ion channel in biological neurons. The dual‐gating geometry provides full tunability of the Gaussian transfer curve, thus enabling the simplified circuit to exhibit a variety of neuronal spike responses, including phase spikes, delayed spikes, and tonic bursts. By utilizing dual‐gated programmability with independent and bias‐dependent biasing, eight different biological neuronal responses can be realized. These results illustrate that adjusting the bias voltage of the modulation terminal can change the function of the device, thus allowing it to data flow and enhance its computing flexibility.

#### 4.1.3 Higher‐Dimensional Functionality

The output characteristics of a multimodal neuromorphic device are mathematically a multivariate function, which means that as the number of input terminals increases, the dimensionality of the multivariate function increases, allowing for a higher‐dimensional mapping of the input data. Higher dimensionality not only enables the discrimination of different features and recognition of various objects,^[^ ^224^, ^225^ ^]^ but also facilitates the representation of data from diverse modalities in a unified manner, thereby enabling multimodal mapping, alignment, fusion, and co‐learning. In this part, we introduce higher‐dimensional functionality that differs from synapses and neurons, simulating not the fundamental units of the nervous system, but rather higher‐level structures.

**Figure 12:**
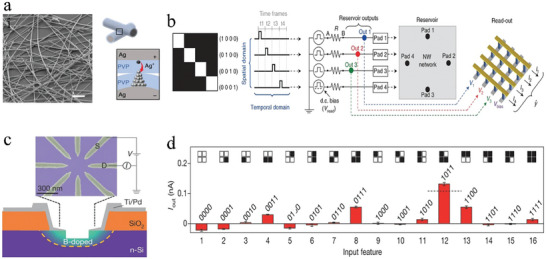
The schematic of higher‐dimensional functionality using homogeneous multimodal devices in electrics. a) Scanning electron microscopy (SEM) image (i) of a highly interconnected memristive NW network reservoir (scale bar, 2 µm) and the representation (ii) of the resistive switching mechanism occurring at the NW junctions. b) Conceptual schematic representation of the experimental implementation of RC in a fully memristive nanoarchitecture where each temporal pattern is applied to different spatial locations of the network. Reproduced with permission.^[^ ^226^ ^]^ Copyright 2022, Springer Nature. c) Schematic of the structure of the silicon‐based system. d) Current response of one of the 16 filters. The 2 × 2 pixel black/white patterns (inset) are represented by ‘0000’, ‘0001’, …, ‘1111’, with black (1) and white (0) mapped to input voltages 0.5 and −0.5 V, respectively. The output current of this filter is maximal when the ‘1011’ pattern is presented. Error bars represent the standard deviation of ten tests. Reproduced with permission.^[^ ^103^ ^]^ Copyright 2020, Springer Nature.

Reservoirs with multiple input terminals can obtain multiple‐dimensional functions through the stochasticity of internal spatial connectivity^[^ ^226^ ^]^ and dynamic processes,^[^ ^227^, ^228^, ^229^ ^]^ realizing high‐dimensional mapping from low‐dimensional input signals, and obtaining the ability of recognition and classification. Milano et al.^[^ ^226^ ^]^ experimentally demonstrated *in materia* RC in a fully memristive architecture based on self‐organized nanowire (NW) networks with random connections among multiple nonlinear elements, as shown in **Figure** **12**a. The emergent NW network dynamics in response to electrical stimulation arise from the interaction of numerous memristive NW cross‐point junctions. The electrochemical potential difference between the crossed NWs induces Ag anodic dissolution to form Ag^+^ ions, which migrate in the polyvinylpyrrolidone (PVP)‐insulated NW shell to form conductive bridges that modulate the junction conductivity.^[^ ^230^ ^]^ The nonlinear dynamics and fading of memory properties (short‐term memory) in the physical reservoir are result from the formation and subsequent spontaneous dissolution of conductive bridges at NW junctions. Pattern recognition was achieved by dividing the 4 × 4 patterns with white (1) or black (0) into four spatial inputs (pattern rows), each containing a stream of four temporal frame inputs (pattern columns). These patterns were then converted into spatio‐temporal inputs connected to the NW network, and the outputs of the NW network were fed toto a classifier constructed from memristive crossbar array, as shown in Figure 12b. In this process, the NW network nonlinearly maps a spatio‐temporal input into a feature space represented by the internal conductive states, such that the input features can be recognized by a simple classification algorithm in a memristive crossbar array. Based on this foundation, the device achieves 90% accuracy in recognizing MNIST handwritten data using a simple classification algorithm, demonstrating the powerful nonlinear mapping capabilities of physical reservoirs to map low‐dimensional data into a high‐dimensional spatial representation, thus obtaining more pronounced differences between various features.

In addition, Chen et al.^[^ ^103^ ^]^ fabricated a multiterminal electrically tunable system consisting of a disordered network of boron dopants in silicon, which exploits the nonlinearity of hopping conduction to mimic filters in the brain and thus perform nonlinear classification and feature extraction (Figure 12c). At sufficiently low doping concentration and temperature,^[^ ^231^ ^]^ the charge carriers are localized, moving sequentially from dopant atom to dopant atom in what is known as the hopping regime. This behavior results in higher resistivity and nonlinearity. In the hopping regime, the potential landscape of this network is highly nonlinearly dependent on the input and control voltages, spanning a high‐dimensional space and mapping low‐dimensional input data to higher‐dimensional output data. For example, this system can perform four‐input binary classification in the form of filtering 16 2 × 2 pixel features. These four pixels are encoded as four input voltages, together with three control voltages and one output current. The network is then evolved into 16 different filters by exploiting the three control voltages, each of which makes one of the 16 features distinguishable from all the others by producing the maximal/minimal output current, thus achieving the map from 4D data to 16D data, as shown in Figure 12d. This demonstrates that high‐dimensional nonlinear mappings can uniformly represent different features in a high‐dimensional space, thus enabling higher accuracy classification and recognition using linear classification methods.

### 4.2 Optics for Bio‐Plausible Eyes

Vision is the primary means by which organisms gather external information, therefore light‐modulated neuromorphic devices have been extensively researched. However, the majority of phototransistors^[^ ^82^, ^125^, ^232^, ^233^, ^234^, ^235^ ^]^ are equipped with only one optical terminal, limiting the light modulation ability on the device, such as the change of synaptic weights along one direction. Inspired by the fact that three or four kinds of cone cells in the retina are sensitive to different wavelengths and thus perceive all visible light, some phototransistors are designed with multiple optical terminals using various light‐sensitive materials. This allows the device to be modulated in a variety of ways by different wavelengths of light.^[^ ^88^, ^89^, ^104^, ^105^, ^127^, ^236^, ^237^, ^238^ ^]^ In this process, light with different wavelengths will activate specific optical terminals to generate carriers, causing them to move in different directions and create a bidirectional photoelectric effect, thereby achieving the bidirectional regulation of the device conductance. Moreover, this working principle simulates the modulation of synapses by neurotransmitter release from a single excitatory/inhibitory neuron in an organism, that is, heterosynaptic plasticity, which is expected to enable multimodal learning.

**Figure 13:**
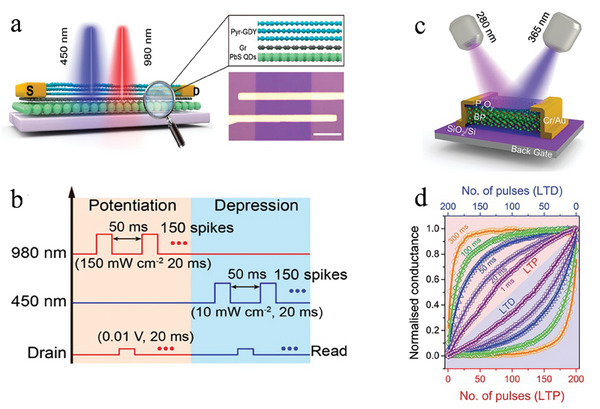
The schematic of heterosynaptic plasticity using homogeneous multimodal devices in optics. a) Schematic of the optical synapse based on the Pyr‐GDY/Gr/PbS‐QD heterostructure and its OM image. Scale bar, 25 µm. b) Schematic of the optical training for LTP/LTD curves. Reproduced with permission.^[^ ^238^ ^]^ Copyright 2021, American Chemical Society. c) Schematic of a BP device fabricated on a SiO_2_/Si substrate. d) Comparison of weight change (conductance) as a function of optical pulse width in 1 to 300 ms range. Reproduced with permission.^[^ ^105^ ^]^ Copyright 2021, John Wiley and Sons.

In 2021, Hou et al.^[^ ^238^ ^]^ proposed a new optical synapse based on a Pyr‐GDY/Gr/PbS‐QDs vertical heterostructure for bidirectional fully‐optical modulation, which provides an optical pathway to control synaptic weights without additional electrical stimulation, simplifying the structure of the synaptic transistor (**Figure** **13**a). Pyr‐GDY and PbS‐QDs in the device are used as the photoresponsive charge trapping layers for inhibitory and excitatory synaptic behaviors, respectively, which enables excitation and inhibition in the optical pathway under the illumination of 980 and 450 nm. Under 450 nm illumination, the Pyr‐GDY layer generates an increased number of electron‐hole pairs. The electrons are subsequently transferred to graphene under the built‐in electric field formed by Pyr‐GDY and graphene, leaving the holes trapped in the Pyr‐GDY layer, which generates a positive photogating effect to reduce the conductance of the graphene layer. In contrast, under 940 nm illumination, the PbS‐QDs layer exclusively produces electron‐hole pairs, inducing a negative photogating effect that increases the conductance of graphene (Figure 13b). These processes simulate the neurotransmitters released from excitatory/inhibitory neurons to regulate synaptic weights, which divides the change of synaptic weights into two processes according to the updating direction that can occur simultaneously and affect each other. This increases bio‐plausibility of the device and holds promising for neural networks with more complex learning rules and higher performance.

Furthermore, the pulse width of the light signal can be used to mimic the amount of neurotransmitter release, which in turn changes the nonlinearity of the synaptic weight changes. Ahmed et al.^[^ ^105^ ^]^ designed an optoelectronic device based on 2D layered black phosphorus (BP), facilitating the realization of a single imaging unit with built‐in memory and signal processing capability due to its fully light‐modulated properties (Figure 13c). The oxidation‐induced defects in vertically‐stacked layers of BP are capable of generating a unique persistent photoresponse under the illumination of UV with different wavelengths,^[^ ^239^ ^]^ so that the programming and erasing operations of the memory are achieved by using light pulses at 280 nm and 365 nm, respectively, thus realizing multi‐bit data programming and storage capability. Under 280 nm illumination, electron‐hole pairs are generated within BP layer, and the electrons are captured by the defective state while the holes stay in the channel, forming a positive and persistent photoconductivity. Whereas, under the irradiation of 365 nm light, the electrons in the defective state are excited to the conduction band, and composite with the holes in the channel, forming a negative photoconductivity effect. These results produce a bidirectional photoconductive effect. In addition, the LTP/LTD characteristics were observed to be correlated to the variation in the width of the light pulse, as shown in Figure 13d. Compared to the short light, the wider could induce relatively significant changes in weight, thus increase the nonlinearity of LTP/LTD and provide new degrees of freedom for device tuning. Using these devices to form neural networks, higher image classification and recognition accuracies (>90%) can be achieved in less than 1000 training cycles, demonstrating their superior learning and computational capabilities.

## 5 Multimodal Neuromorphic Circuit

Sections 3 and 4 provide a review of the diverse functions of heterogeneous and homogeneous multimodal neuromorphic devices, which exhibit superior sensing and computing capabilities.^[^ ^57^, ^91^, ^156^, ^238^ ^]^ Further, by interconnecting them in the form of crossbar arrays, it is possible to obtain a finer‐grained perception of distributed physical signals as well as neural‐network‐like extraction of information for identification, classification, judgment, inference, and decision‐making.^[^ ^91^, ^94^, ^98^, ^119^, ^123^, ^240^, ^241^, ^242^ ^]^ This section will focus on the circuit and application of multimodal neuromorphic devices, which can function independently as fundamental units or be integrated with other sensors in circuits.

**Figure 14:**
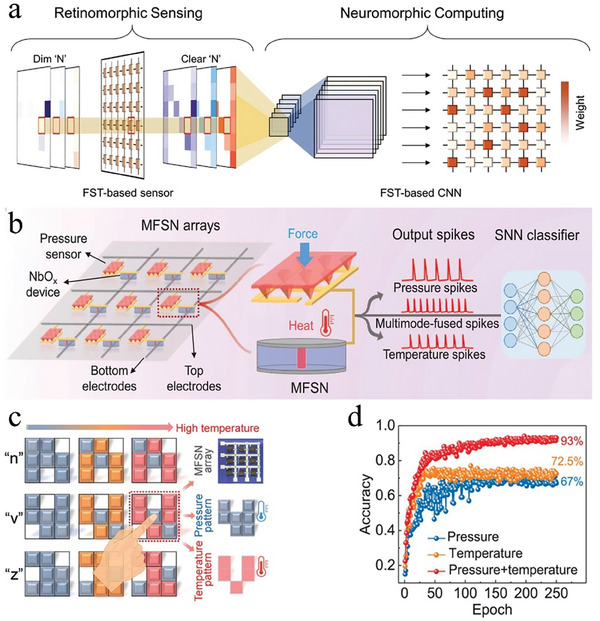
The schematic of multimodal neuromorphic device integration. a) Schematic of the ferroelectric transistors‐based NMVS. It contains a sensor array for retinomorphic sensing and CNNs for neuromorphic computing. The sensor can respond to light with a wavelength ranging from DUV (275 nm) to NIR (808 nm) and has a visual adaption behavior and a large dynamic range. Reproduced with permission.^[^ ^133^ ^]^ Copyright 2023, John Wiley and Sons. b) Artificial somatosensory system consisting of an MFSN array and an SNN classifier to emulate tactile perception. c) Schematic diagram of three raised objects (“n,” “v,” and “z”) with pressure noises perceived by a human hand at three different temperatures (20, 40, and 60 °C indicated in cyan, orange, and pink, respectively). The three small images from top to bottom on the right side show an optical image of the 3 × 3 MFSN array, a pressure pattern, and a temperature pattern, respectively. d) Evolution of the training accuracy with training epoch at three different modes. Reproduced with permission.^[^ ^56^ ^]^ Copyright 2022, John Wiley and Sons.

The integration of individual multimodal neuromorphic devices into arrays can endow the system with the ability to finely sense and store physical objects,^[^ ^240^, ^241^ ^]^ saves the whole physical object in arrays, and utilizes electrical modalities as a bridge for information transfer and processing between devices. Lee et al.^[^ ^242^ ^]^ deposited a layer of InGaAs as a photoreceptor on an HfO_2_ RRAM, where each photoreceptor cell acts as a pixel to store the complete image data in the arrays and is capable of performing edge computation of handwritten digits by VMM. Wang et al.^[^ ^98^ ^]^ integrated flexible synaptic transistors with thermal, mechanical, and electrical modalities into arrays, demonstrating thermal‐ and strain‐tunable synaptic plasticity as well as tunable temperature‐position resolution, therefore efficiently monitoring temperature and/or strain changes in real‐time. As a result, the integrated arrays exhibit sensing, transmission, memory, and recovery of external stimuli akin to that of human skin. In addition, multimodal neuromorphic device arrays can utilize the superior sensing and computational capabilities of individual devices to build neural networks with fine‐grained sensing capabilities. For instance, Cai et al.^[^ ^133^ ^]^ proposed a complete neuromorphic machine vision system (NMVS) based on α‐In_2_Se_3_ ferroelectric transistors, which demonstrate electrical and optical modalities capable of integrating front‐end retinomorphic sensors and a back‐end convolutional neural network (CNN) into the same arrays, as shown in **Figure** **14**a. Due to the electro/photo‐induced ferroelectric polarization of α‐in_2_Se_3_, the device can simultaneously sense light signals and utilize electrical signals to compute. Furthermore, the device exhibits a nonlinear optical response of retinal‐like visual adaptive function and linear long‐term plasticity under electrical modulation. Thus, multilayer arrays fabricated using this device show biologically comparable optical sensing and electrical computation capabilities, realizing a compact multilayer neural network with sensing capabilities and image recognition accuracies of up to 93%, which is 20% higher than that of an incomplete system without front‐end retinomorphic sensors. This indicates that the construction of neural networks using neuromorphic devices capable of multimodal fusion has the potential to significantly improve recognition accuracy.

By utilizing the electrical terminal of a multimodal neuromorphic device as a coupling port to connect other physical sensors, the system can enhance its capability to detect a wider range of more detailed physical signals. In 2022, Zhu et al.^[^ ^56^ ^]^ reported an artificial multimodal sensory system consisting of a multimodal fusion spiking neuron (MFSN) array. The array is composed of a piezoresistive pressure sensor and a NbO_x_‐based Mott memristor, and a spiking neural network (SNN) classifier for achieving human‐like multisensory perception (Figure 14b). As the pressure increases, the resistance of the piezoresistive sensor decreases, leading to an increase in the rate of spikes in the output of the MFSN unit. With an increase in temperature ranging from 20 to 65 °C at a constant voltage, the threshold voltage of the Mott memristor decreases, resulting in a decrease in amplitude and an increase in spiking rate. As a result, the device is capable of detecting both pressure and temperature, and it differentiates between the two based on spike amplitude and frequency. Figure 14c shows a schematic of human hand touching “n”‐, “v”‐, and “z”‐shaped objects at different temperatures, where the shapes of the objects touched are mapped to patterns formed by discrete weights on sensors, and the output is extracted from the decoupled frequency and amplitude features, forming a 6 × 3 matrix for recognizing hand touching data. Experimental evidence indicates that the accuracy of pattern recognition (95.6%) using both mechanical and thermal modal information (pressure‐dependent frequency (F_p_) and temperature‐dependent amplitude (A_T_)) is superior to that of using pressure information (F_p_,89.9%) and temperature information (A_T_,91.1%), respectively, at the same ambient temperature (20 °C). Furthermore, recognition accuracy (40 °C, 97.8% and 60 °C, 100%) improves as the ambient temperature increases. In addition, an object classification work was performed to demonstrate the feasibility of the MFSN in practical applications. Eight frequency patterns were collected from a simulated 20 × 20 MFSN array that represents eight cups of different shapes, temperatures, and weights. The simulation results reveal that the pattern with multimodal perception exhibits a higher recognition rate (93%) in distinguishing cup features compared to the single‐modal pattern using only temperature (72.5%) or pressure (67%) for tactile perception (Figure 14d), suggesting multimodal data can provide a more reliable basis for recognition and judgment.

## 6 Conclusion and Outlook

Multimodal learning is a prevalent and crucial capability of organisms, enabling them to integrate and utilize multisensory information for adaptation to their environment. Inspired by this capability, multimodal machine learning has been proposed to empower artificial systems with bio‐plausible abilities to automate complex tasks. However, the perception and representation of multimodal information pose significant challenges for hardware and software architectures in machine learning. Multimodal neuromorphic devices can achieve the perception and representation of multimodal information through hardware. The regulation of complex and diverse physical signals to the device is finally transformed into the electrical output of the device, providing a physical foundation for further processing and application of multimodal information. In this review, we have summarized the recent advances in multimodal neuromorphic devices with multi‐physical sensing and multi‐signal processing capabilities. Based on the variations in physical signals and the specific focus of their capabilities, we categorized the multimodal neuromorphic devices into heterogeneous ones prioritizing sensing various physical signals and homogeneous ones focusing on processing multiple identical physical signals and elaborated their functions in terms of physical mechanisms and computational capabilities, respectively. Multimodal neuromorphic devices integrate information from diverse modalities into electrical signals, providing a physical basis for subsequent modality alignment, mapping, fusion, and co‐learning. Integrating these multifunctional devices in a distributed network structure, that is, a crossbar array, enables higher dimensional computational and fine‐grained perceptional capabilities, thereby empowering the system to recognize, judge, reason, and make decisions. This approach improves the accuracy of traditional architectures for recognition and classification, and enhances the perception of dynamic signals to achieve tasks that are difficult to be achieved with traditional device arrays.

Despite the bright prospects, it is of importance to carefully consider the impediments and complexities encountered in the investigation of multimodal neuromorphic computing:

1. Reliability and variability. The device is equipped with multiple terminals coupled to various physical signals. Volatile changes induced by the signals introduce noise in the internal states of the device, while non‐volatile changes result in variability from cycle to cycle and device‐to‐device. Reliability challenges in complex and changeable environments, where the device state is affected by noise from different terminals, requiring a comprehensive understanding of the internal physical processes to reduce the impact of noise on the device state.
2. Integrated architecture and scalability. Integrating multifunctional devices into a traditional crossbar array does not fully utilize device's performance and thus requires a trade‐off between individual device performance and system complexity. An increase in the number of devices in the array, or array size, has the potential to enhance the system's fine‐grained sensing and information extraction capabilities. However, this is not a universal solution, as it is not applicable to all device architectures. The design of specialized neural network architectures based on specific device architectures,^[^ ^243^ ^]^ or the unified simplification of device architectures to align with existing architectures, can facilitate the realization of the full potential of the device.
3. Algorithm design. The lack of theoretical guidance at the algorithmic level for the design of multimodal neuromorphic devices and multimodal neuromorphic computational systems, and the need to design corresponding algorithms for specific multimodal neuromorphic systems have hampered parallel and simultaneous development of software and hardware.^[^ ^244^ ^]^ Thus algorithm‐hardware co‐design is essential for the development of multimodal neuromorphic computing.

Overall, the preparation, regulation, and integration of high‐performance multimodal neuromorphic devices require an in‐depth understanding of the intrinsic mechanism to establish a response‐mechanism correspondence. This involves drawing upon neuroscience theories and research findings to optimize the physical interconnections between devices, as well as developing corresponding algorithms to co‐design the multimodal architectures. In device preparation, selecting materials with rich physical responses (e.g., 2D ferroelectric semiconductor α‐In_2_Se_3_), or stacking different functional materials together through heterogeneous integration technology is conducive to the realization of multimodal neuromorphic devices. Furthermore, by gaining a comprehensive understanding of the physical processes and implementing material modifications, it is possible to correlate different physical signals with various state variables. This allows for the decoupling of physical signals in order to enhance reliability. In system connectivity, inspired by biological systems, it is possible to fabricate complex networks architecture compatible with multiterminal devices, such as dendritic network,^[^ ^243^ ^]^ thereby unlocking device spatio‐temporal information processing capabilities to improve system's scale and realizing high‐performance artificial networks that conform to biological benchmark. In the algorithm design, the top‐down demand for the hardware system can be theoretically guided by the hardware system implementation and performance enhancement, realizing the algorithm and hardware co‐design and common development.^[^ ^244^ ^]^ It is foreseeable that multimodal neuromorphic devices with multi‐physical sensing and multi‐signal processing capabilities will provide artificial systems with enhanced intelligence, diversity, and adaptability to the external environment, thereby driving the advancement of the next generation of artificial intelligence.

## Conflict of Interest

The authors declare no conflict of interest.
